# Targeting the Immune Microenvironment in Lymphomas of B-Cell Origin: From Biology to Clinical Application

**DOI:** 10.3390/cancers11070915

**Published:** 2019-06-29

**Authors:** Tom A. Mulder, Björn E. Wahlin, Anders Österborg, Marzia Palma

**Affiliations:** 1 Department of Oncology-Pathology, Karolinska Institutet, 171 76 Stockholm, Sweden; 2 Department of Hematology, Karolinska University Hospital, 171 76 Stockholm, Sweden

**Keywords:** B-cell lymphoma, tumor microenvironment, immune cells, T cells, PD-1, PD-L1, macrophages, CD47, Hodgkin lymphoma

## Abstract

In lymphomas of B-cell origin, cancer cells orchestrate an inflammatory microenvironment of immune and stromal cells that sustain the tumor cell survival and growth, known as a tumor microenvironment (TME). The features of the TME differ between the different lymphoma types, ranging from extremely inflammatory, such as in Hodgkin lymphoma, to anergic, leading to immune deficiency and susceptibility to infections, such as in chronic lymphocytic leukemia. Understanding the characteristic features of the TME as well as the interactions between cancer and TME cells has given insight into the pathogenesis of most lymphomas and contributed to identify novel therapeutic targets. Here, we summarize the preclinical data that contributed to clarifying the role of the immune cells in the TME of different types of lymphomas of B-cell origin, and explain how the understanding of the biological background has led to new clinical applications. Moreover, we provide an overview of the clinical results of trials that assessed the safety and efficacy of drugs directly targeting TME immune cells in lymphoma patients.

## 1. Introduction

Lymphomas are a heterogeneous group of cancers arising from lymphocytes, typically involving lymphoid organs. Many subtypes exist, defined by the current World Health Organization (WHO) classification primarily based on tumor cell morphology, immune phenotype and genetic alterations [1]. The cell of origin, identified based on the microscopic appearance and immune phenotype of the tumor cells, is the basis for subtyping. Two main groups are defined: B-cell lymphomas, and T-cell and natural killer (NK)-cell lymphomas. Hodgkin lymphoma represents an independent entity, but, given its B-cell origin, it is discussed together with B-cell lymphomas here. Lymphomas account for approximately 3.5% of new cancer cases worldwide, with B-cell lymphomas and Hodgkin lymphoma accounting for 80% and 10% of all lymphoma cases, respectively [2].

In general, it is assumed that the major oncogenic genetic events accumulate prior to or at the cell-of-origin stage of B-cell development and contribute to hampering further maturation [3]. The presence of somatic hypermutation (SHM) allows us to determine whether the lymphoma cell has experienced the germinal center (GC) or not. The presence of SHM in both primary mediastinal large B-cell lymphoma (PMBCL) and classical Hodgkin lymphoma (cHL), for example, supports the GC or post-GC origin of these lymphoma types. Mantle cell lymphoma (MCL) cells, instead, have features that are evocative of naïve B cells, even if SHM is found in one third of the cases [4], while Burkitt lymphoma (BL), follicular lymphoma (FL) and GC B-cell (GCB) diffuse large B-cell lymphoma (DLBCL) have features that are suggestive of GC B-cell derivation. The activated B cell-like (ABC) DLBCLs are also GC-experienced, but the cell of origin in this case is suggestive of plasmablast cell differentiation. Marginal zone lymphoma (MZL) cells have a microscopic appearance and immune phenotype that is reminiscent of B cells from the marginal zone of the B-cell follicles. The cell of origin in chronic lymphocytic leukemia (CLL), on the other hand, is still a matter of debate. According to initial theories, CLL cases with mutated immunoglobulin (Ig) heavy-chain variable region genes (IGHV) would derive from a GC-experienced B cell while cases with unmutated IGHV would derive from a naive B cell, but this issue is still widely disputed [5].

In the past decades, lymphoma pathogenesis has been better understood, with increasing focus put on non-malignant cells residing in the tumor, primarily immune and stromal cells, which constitute the so-called tumor microenvironment (TME) [6]. This knowledge has provided the rationale for new clinical applications and therefore the opportunity to address unmet clinical needs.

In this review, we summarize the available pre-clinical data on the role of the immune cells in the TME and provide an overview of the clinical trials with drugs targeting TME immune cells in lymphoma patients.

## 2. Immune Cells in the Tumor Microenvironment

Lymphoma TME can vary substantially among the different lymphoma types. A summary of the key pathological features of the different lymphomas of B-cell origin, including the TME composition, is provided in Table 1.

The TME composition somehow mirrors the degree of dependence of tumor cells on TME cells for survival and proliferation. Indeed, the cancer cells can actively shape the TME composition by, for example, expressing chemokines and adhesion molecules that attract certain immune cells rather than others, or favor their homing to the lymph nodes rather than extra-nodal dissemination. Scott and Gascoyne defined three extreme patterns of TME composition, named ‘re-education’, ‘recruitment’ and ‘effacement’, which are exemplified by FL, cHL and BL respectively [3]. Beside these major patterns, variable degrees of interplay between malignant cells and immune cells in the TME are observed in the different lymphoma subtypes.

Besides sustaining tumor cell survival and growth, these interactions also contribute to the inhibition of an effective anti-tumor immune response by various mechanisms. The overexpression of the so-called immune checkpoints, co-signaling receptors that participate in the regulation of T cell-driven immune responses, is one of these mechanisms. Programmed death-1 (PD-1) is an immune checkpoint which inhibits T-cell functions upon binding to the ligands PD-L1 and PD-L2 [7]. PD-L1 is lowly expressed in normal tissues, but highly expressed on various tumors and can be further enhanced by tumor environmental factors [8,9]. Strategies aiming at unleashing anti-tumor immunity by blocking these immune checkpoints, including the so-called immune checkpoint blockade (ICB), has been implemented in cancer treatment during the past decade.

### 2.1. Diffuse Large B-Cell Lymphoma

This subtype is the most common B-cell lymphoma, accounting for 30% of all lymphomas [10]. In general, DLBCL is recognized by a diffuse growth pattern of large CD20^+^ cells, with additional immune phenotypes varying among the different subtypes [11]. By comparing the transcriptional profiles of primary DLBCL cells with healthy B cells, two distinct genetic subgroups were identified suggesting the initiating cell type: DLBCL arises from GC and post-GC B cells. Regarding the gene expression profile, the GCB-DLBCL subtype resembles the GC cells and the ABC-DLBCL subtype is reminiscent of in vitro-activated B cells and of plasma cells, with the latter subtype having a much poorer prognosis [12].

Typically, the tumor cell content is 60%–80% [13], corresponding to a rather ‘effaced’ TME composition pattern, as proposed by Scott and Gascoyne [3]. The tumor cells have acquired genetic mutations that render them relatively independent from survival and proliferation signals from their microenvironment [3]. Immune cells in the TME of DLBCL include natural killer (NK) cells (+/−20% of total cell content), dendritic cells (DCs) (+/−15%), M2-type macrophages (+/−15%), CD4+ T cells (+/−10%) and CD8^+^ T cells (< 5%) [13].

The contribution of monocyte-derived cells, such as tumor-associated macrophages (TAMs) and myeloid-derived suppressor cells (MDSCs), to the pathophysiology of DLBCL and their prognostic impact remain highly controversial [14]. Two general studies in B-cell non-Hodgkin lymphoma (NHL) show that patients with increased ratios of circulating CD14^+^HLA-DR^low/−^ monocytes have more aggressive disease and suppressed immune functions and that the expansion of this population may be mediated by interleukin (IL)-10 [15,16]. Interestingly, CD47, a surface molecule that inhibits cellular phagocytosis, is upregulated on primary DLBCL cells and predicts poor prognosis [17]. CD47 also enables extranodal dissemination. Targeting this protein with an agonistic antibody could prevent dissemination and induce tumor cell phagocytosis [17,18].

In the TME of DLBCL, T cells have emerged as good tools for predicting prognosis and they possibly play a large role in lymphomagenesis. Low general T-cell infiltration correlates with poor survival in DLBCL [19,20,21,22]. Both a high percentage (> 6%) of tumor infiltrating CD8^+^ T cells [23] and a high CD4^+^ T-cell infiltration have been correlated to increased survival [24]. These CD4^+^ tumor-infiltrating lymphocytes (TILs) were mostly CD4^+^CD45RO^+^ memory T cells [24,25]. The prognostic value of a high CD4:CD8 ratio among tumor-infiltrating T cells remains unclear, as it has been associated with both better and worse survival in different studies [21,22]. On the other hand, no significant prognostic correlation has been found with regulatory T cells (Tregs) [19,26,27].

Through gene set enrichment analysis, 3 subsets of DLBCL were identified, among which the ‘host response’ (HR) subset. These HR tumors had increased expression of immune effector cell markers and higher numbers of CD3^+^ TILs and interdigitating S100^+^ DCs [28]. These findings were confirmed in a study where S100^+^ DCs were found together with CD45RO^+^ T cells in the tumor periphery correlating to increased numbers of granzyme B^+^ TILs [29]. Interestingly, macrophage and CD8^+^ T-cell infiltration in the bone marrow is more common in high-risk DLBCL patients and CD8^+^ T-cell bone marrow infiltration is a negative prognostic indicator independent of bone marrow involvement [30]. Furthermore, CD40 expression on tumor cells correlated with a longer overall survival (OS), suggesting that the interaction between tumor cells and CD40L^+^ CD4^+^ T cells could stimulate antigen presentation and lymphoma-specific T-cell responses [31].

The importance of T-cell infiltration in the prediction of patient outcome in this disease suggests a potential benefit of treatment with ICB. The expression pattern of CTLA-4 in the DLBCL TME is understudied, but it is thought to play a role in T-cell priming rather than peripheral tolerance. Non-GC DLBCL in particular has a high expression of immune escape genes and immune checkpoint molecules in the TME [32]. Overall, PD-L1 expression on tumor cells has been reported in only a subset of DLBCL patients, mainly in those with a non-GC subtype and Epstein-Barr virus (EBV)-associated disease [33,34,35,36,37]. Genetic alterations in the PD-L1/PD-L2 locus are a common cause of overexpression [38]. PD-L1 is also expressed by macrophages in the DLBCL TME and the infiltration of PD-1^+^ lymphocytes was associated with a superior survival in a cohort of patients receiving standard chemo-immunotherapy treatment (R-CHOP; rituximab, cyclophosphamide, doxorubicin, vincristine and prednisone) [39]. Both tumor-specific PD-L1 expression and soluble PD-L1 correlate with poor prognosis [40,41,42]. Tissue PD-L1 expression, PD-1 expression on T cells and PD-1/PD-L1 interaction are negative prognostic indicators in patients with high T-cell infiltration, i.e., >3.5% of all cells [20].

Primary mediastinal large B-cell lymphoma is an official subtype of DLBCL, although it shares more histopathological and gene expression patterns with the nodular sclerosis subtype of cHL [11]. In PMBCL, frequent rearrangements (20% of cases) of the PDL locus 9p24.1 have been found, that lead to increased expression of PD-L1, PD-L2 and Janus Kinase (JAK)2 transcripts [33,34,37,43,44,45]. Overexpression of JAK2 could further enhance overexpression of PD-L1 [46].

The composition of the TME is slightly different in DLBCL cases that occur at the so-called immune-privileged sanctuary sites. Compared to nodal DLBCL, primary central nervous system (CNS) lymphoma (PCNSL) has a lower infiltration of S100^+^ DCs and cytotoxic T cells (poor prognostic indicator). The presence of infiltrating S100^+^ DCs was correlated with infiltrating T cells in a rimming pattern [47]. Low expression of a T-cell gene signature and a low infiltration of CD4^+^ and CD8^+^ predict inferior survival in primary testicular lymphoma (PTL) [48]. PD-L1 is frequently expressed on tumor cells and macrophages in the TME of PCNSL [49]. Copy number alterations and translocations involving the 9p24.1 locus, as a basis for PD-L1 overexpression, are also common in PCNSL and PTL [50,51].

Besides expression of immune suppressive molecules on T cells, ineffective antigen presentation by tumor cells can also contribute to hampering anti-tumor immune responses. B-cell lymphomas usually express major histocompatibility complex (MHC) class II molecules, reminiscent of their cell of origin. However, there is ample evidence of antigen presentation and immune recognition defects in DLBCL. CD4^+^ T cells with cytotoxic capabilities occur in DLBCL and their presence correlates with the MHC class II expression by tumor cells. Even though these T cells can kill DLBCL cells in vitro in an MHC class II-dependent manner, no correlation was found with patient survival [52]. In an extensive immunohistochemistry study, the loss of CD86, CD54, MHC class I and II expression on DLBCL cells were all associated with low tumor infiltration by CD8^+^ T cells [53]. Moreover, loss of MHC class II expression on DLBCL tumor cells correlates with poor patient survival and lower numbers of TILs [54,55,56].

In particular, PCNSL and PTL cases have commonly lost MHC class I and II expression, frequently caused by homozygous deletions in the MHC class II genes [57]. Loss of MHC class I and II expression in PTL was also found to correlate with low T-cell infiltration [48].

The loss of MHC class II expression in nodular DLBCL cases, however, is not commonly caused by deletions, suggesting transcriptional or post-transcriptional regulation [58]. Decreased class II transactivator (CIITA) expression emerged as the most common mechanism of MHC class II downregulation [59]. Normal B cells physiologically lose MHC class II expression through plasma cell differentiation. Accordingly, MHC class II expression is high in GCB-DLBCL, low in ABC-DLBCL and negative in plasmablastic lymphoma (PBL). An inverse correlation exists between the expression of MHC class II and plasma cell markers. These observations collectively suggest physiological plasma cell differentiation as another mechanism of downregulation and loss of MHC class II expression in DLBCL [60]. In PMBCL, decreased MHC class II expression is related to poor patient survival [61]. In this DLBCL subtype, genomic rearrangements in CIITA occur frequently, which correlates with a shorter disease-specific survival. CIITA gene fusions lead to reduced MHC class II surface expression and overexpression of PD-1 ligands PD-L1 and PD-L2 [62,63].

Other molecular mechanisms contributing to defective antigen presentation have also been suggested. The expression of gamma-interferon-inducible lysosomal thiol reductase (GILT), a facilitator of endocytic binding of peptides to MHC class II, correlates with better patient survival [64]. Besides, in an in vitro model for ABC-DLBCL, FOXP1 has been shown to reduce the expression of MHC class II and CD74 [65].

Inactivating mutations and deletions in the β2-Microglobulin (B2M) gene, crucially impairing MHC class I assembly and cell surface expression, occur in 29% of DLBCL cases. The lack of MHC class I helps tumor cells escape from recognition by CD8^+^ cytotoxic T cells, yet trigger recognition by NK cells. However, concurring genetic lesions in CD58 occur in 21% of cases, aiding escape from NK cell-mediated cytotoxicity. A mere 61% of DLBCL cases lack both MHC class I and CD58 surface expression [66].

Several targetable epigenetic regulators and other enzymes interfere with the antigen presentation machinery in DLBCL. In GCB-DLBCL cells, CIITA transcription is epigenetically regulated by histone deacetylases (HDAC) and HDAC inhibitors can reverse this process [67]. cAMP response element binding protein (CREB) binding protein (CREBBP) mutant GC lymphomas have reduced MHC class II expression through epigenetic silencing by HDAC3 [68,69]. Moreover, EZH2, an epigenetic repressor, is frequently mutated in both MHC class I- and II-negative GC B-cell lymphomas. MHC expression can be restored by the EZH2 inhibitor tazemetostat in vitro [70]. Furthermore, an inhibitor of the protein mucosa associated lymphoid tissue (MALT)1, a paracaspase crucial in B-cell activation, suppresses the growth of ABC-like DLBCL cells in vitro and in vivo. MALT1 drives JAK/signal transducer and activator of transcription (STAT) signaling and inhibits type I interferon and MHC class II expression [71]. Besides, MALT1 facilitates PD-L1 expression on ABC-DLBCL cells [72]. Interestingly, MHC class II was lost and PD-L1 was upregulated in a nuclear factor kappa-light-chain-enhancer of activated B cells (NFκB)-driven and p53-deficient ABC-like murine DLBCL model. PD-1 blockade enhanced the efficacy of anti-CD20 antibody treatment in this immunocompetent ABC-DLBCL model [73].

### 2.2. Follicular Lymphoma

The development of the indolent lymphoma subtype FL is linked to normal B-cell development, hence the FL cell retains features from its normal progenitor, the GC B cell, such as somatic hypermutation (SHM) and CD10 expression [74]. Like the GC B cell, the FL cell is completely dependent on its microenvironment and therefore cell lines have been difficult to establish [75]. When FL spreads to the bone marrow, it appears to both recruit T-cell subsets and train local stroma cells to nurture it [76]. Very common neoplastic changes in FL cells are: t(14;18)(q32;p21), leading to overexpression of B-cell lymphoma 2 (BCL2) [74], glycan modification of the B-cell receptor (BCR) [77] and mutations in epigenetic regulators [78].

Normally, only GC B cells selected for their BCR will survive and differentiate, while the others will go into apoptosis. However, the aberrant and abundant expression of BCL2 protects FL cells from this destiny. Upon recognition of an antigen, the BCR launches important downstream survival signals in a normal B cell. It is noteworthy that FL cells always express the BCR (with its surface Ig), even though one allele has been consumed by the BCL2 translocation. This suggests that FL needs the BCR to survive. In idiotype-vaccine experiments, resistance in FL arose through new mutations in the Ig gene, not through loss of BCR/Ig expression [79,80]. In about a fourth of the cases, the BCR in FL recognizes auto-antigens, such as vimentin [81,82]. However, most FLs appear to be dependent on BCR signaling without any extant antigen. In over 80% of cases of FL, somatic mutation has introduced N-glycosylation in the antigen-binding Ig variable region of the BCR [77]. These added mannoses interact with lectins of the mannose receptor and CD209, expressed by cells in the microenvironment, and ultimately, escape the normal BCR selection [83]. Almost-always present are one or several mutations that cause deregulation of the epigenome (reviewed extensively in reference [78]), impairing the FL cell’s ability to differentiate beyond the GC phenotype. Additionally, the 60%–70% of FLs with CREBBP mutations decrease MHC class II expression, associated with a reduced frequency of various infiltrating T-cell subsets [84].

The immune microenvironment in FL partly resembles the normal GC B-cell environment, with the FL cell (like the GC B cell) being dependent on follicular helper T cells (TFHs) and follicular dendritic cells (FDCs). Macrophages are more common in FL than in normal GCs and they express lectin, possibly upregulated by IL-4, which also stimulates FL survival through the BCR [85]. IL-4 is highly expressed in the FL milieu [86], produced by local TFHs [87]. The abnormally high production of IL-4 by FL TFHs has been shown to be induced by FDCs and fibroblastic reticular cells, through the overexpression of stromal cell-derived factor-1 (SDF-1, also known as CXCL12) [88]. The normal function of the chemokine CXCL12 is to polarize the GC to dark (centroblast) and light (centrocyte) zones [89], with the CXCL12-receptor CXCR4 highly expressed in centroblasts but not in centrocytes [90]. The effect of CXCL12 in FL appears to be mostly mediated through CXCR4^+^ TFHs. The TFHs also express CD40L, another important survival signal for FL [91]. FDCs provide a scaffold and secrete besides CXCL12 also CXCL13, which attracts FL cells and TFHs through ligation of CXCR5, to make them move around the FDC [92]. The FL cells seem to trigger the recruitment, polarization, and maintenance of their microenvironment, with the exact mechanisms yet to be uncovered. For example, patients with FL involvement of the bone marrow, which occurs in about 50% of the cases, have a deranged bone marrow microenvironment, which is not found in patients without bone marrow involvement. However, patients who will later develop bone marrow involvement already show a deranged bone marrow environment at diagnosis, in which the bone-marrow FL-cells have recruited CD4^+^ and PD-1^+^ T cells at the expense of CD8^+^ T cells, and have subverted local stromal cells to an CD21^+^ FDC phenotype [93].

Intratumoral CD4^+^ T cells that have a high PD-1 expression are mainly found in the lymph node follicles, have a TFH phenotype, are CXCR5^+^, BCL6^+^, TIM-3^-^, secrete IL-21 and support the proliferation of B cells. The CD4^+^ T cells that have a low PD-1 expression, in contrast, are arranged in an interfollicular pattern, do not express CXCR5 and BCL6, yet do express exhaustion marker TIM-3. Furthermore, these CD4^+^ or CD8^+^ PD-1^low^ T cells correlated with inferior OS [94]. High tissue PD-1 expression has been associated with good clinical outcome [95], though PD-1 expression has also been correlated to inferior patient survival [96,97,98].

Numerous studies have been published on the prognostic properties of immune cell subsets in the microenvironment of FL, but their prognostic values have been difficult to reproduce [92]. This difficulty is aggravated by the enormous survival improvements in the last decades, and the median OS in this indolent disease is now at least 15 years [99,100,101]. The changing therapeutic paradigms (most importantly, the advent of rituximab) have probably also changed the prognostic properties of the immune cells in the TME since the effect of rituximab is partly dependent on some immune cells. Finally, it might be possible that circulating immune cells are more important than local TME cells when rituximab is used [102].

### 2.3. Chronic Lymphocytic Leukemia

Chronic lymphocytic leukemia is the most common leukemic lymphoma, and is characterized by the presence of lymphocytosis (≥5.0 × 10^9^/L) and a typical immune phenotype of clonal B cells that express CD5, CD19 and CD23. The disease is incurable, with a highly variable clinical course, ranging from indolent and not requiring treatment, to active, treatment-requiring disease due to progressive marrow failure, symptomatic lymphadenopathy, splenomegaly or disease-related symptoms. Typically, the majority of CLL cells are in a resting state. The anatomical site of CLL cell proliferation is thought to be the so-called proliferation centers in the lymph nodes [103].

The TME of CLL is complex and multidimensional. Relevant interactions between immune cells and tumor cells take place in all the tumor compartments, yet mainly in the lymph nodes and the bone marrow and to a lesser extent in the peripheral blood. CLL cells and immune cells in the TME exert a reciprocal influence on each other. Monocytes, macrophages and T cells are the immune cells most commonly found in the TME, in very variable frequencies.

Monocyte-derived nurse-like cells (NLCs) attract CLL cells in vitro through the secretion of the CXCR4 ligand CXCL12 [104], and the CXCR5 ligand CXCL13 [105]. NLCs can be generated from monocytes in vitro by co-culturing them with CLL cells, potentially mediated by CLL-secreted HMGB1 [106] and M-CSF [107]. These in vitro-generated cells upregulate the TAM markers CD68 and CD163 in vivo [108] and patients with high serum levels of soluble CD163 have shorter treatment-free survival and OS [109]. Genes that are specifically altered by CLL cells in these monocyte-derived cells are involved in the ability to process and present antigens [110]. CLL cells receive survival cues from NLCs, which rescues them from spontaneous apoptosis in vitro and drug-induced apoptosis in vivo [111]. The survival signals to CLL cells include, but are not limited to, the expression of CXCL12 [104], CD31, plexin B [112], a proliferation-inducing ligand (APRIL), B-cell-activating factor (BAFF), [113], BCR stimulation [114] and CD2 expression [115] by NLCs. These interactions have recently been reviewed by ten Hacken and Burger [116]. Ligation of the chemokine receptors CXCR4 and CXCR5 (by CXCL12 and CXCL13, respectively) leads to endocytosis and down-regulation of their surface expression [117,118]. Antigenic stimulation of the BCR makes CLL cells migrate towards CXCL12 and CXCL13, secrete CCL3 and CCL4 (T cell-attracting chemokines) and downmodulate CXCR4 expression. These BCR-dependent mechanisms that create the protective niche for CLL cells could be abrogated downstream (such as by a Syk-inhibitor in vitro [119]).

The protection by NLCs is akin to the role of bone marrow stromal cells (BMSCs) [120,121], where CXCR4 is also an important chemoattractant for CLL cells [122], yet they evoke distinct gene expression profiles in CLL cells. NLCs activate expression of genes within the BCR and NFκB signaling pathways [114], while BMSCs upregulate TCL1 and FOS/JUN expression [123].

It is well established that CLL cells are inadequate antigen-presenting cells (APCs) [124,125]. Additionally, viral and bacterial infections are a common cause of disease-related morbidity, which displays an acquired deficiency of the adaptive immune system [126,127]. Peripheral blood CD4^+^ T cells have defective helper functions and suppressive activity [128] and CD8^+^ cells have substantial reduction in CD3 zeta chain and CD28 expression [129]. Compared to healthy controls, T cells in CLL patients have downregulated genes encompassing cell differentiation (CD4^+^), cytoskeleton formation, vesicle trafficking and cytotoxicity (CD8^+^). Similar gene expression profiles could be provoked in healthy donor T cells through co-culture (in direct contact) with CLL cells [130]. T cells are suppressed by CD200 expression on the surface of CLL cells [131]. Furthermore, circulating CD4^+^ and CD8^+^ have a ‘pseudoexhausted’ phenotype [132,133,134], with upregulation of BLIMP1, CD160, CD244, and PD1, most notably in effector T cells. CD8^+^ T cells have deficient proliferation and cytotoxicity, caused by defective vesicle packaging of granzyme B and faulty formation of immune synapses with non-polarized degranulation [135,136]. CD200, B7-H3, HVEM and PD-L1 were all shown to mediate the development of immune synapse defects [137]. CLL cells exhibit reduced motility, which is crucial for immune synapse formation, through inhibition of Rho GTPase signaling [138]. Immune synapses between APCs and T cells in CLL were reported to be restored by the immune modulating drug lenalidomide [136,137,138]. Interestingly, inferior OS was associated with increased PD-1 expression by T cells in the lymph nodes [137].

Increased frequencies of Tregs (10% of total CD4^+^ T cells) have also been reported in the peripheral blood of CLL patients, especially in advanced disease [134,139].

In the tissue niches where CLL cells proliferate, T cells play a major role too [140]. CCL22 is secreted by CLL cells in the bone marrow and lymph nodes, yet not by those in the peripheral blood. This chemokine attracts CD4^+^ T cells expressing the CCL22 receptor CCR4 and CD40L that support survival [141,142]. Typically, CLL cells proliferate in ‘pseudofollicles’, where they are expressing high levels of CD38 and are in tight contact with CD4^+^ T cells [143]. Furthermore, CLL cells secrete the T-cell chemokines CCL3 and CCL4 upon BCR stimulation by NLCs, so NLCs indirectly contribute to the attraction of chronically activated (CD57^+^) T cells to these protective tumor niches [114,144]. CCL3 and CCL4 levels are also elevated in CLL patients’ plasma [114,145]. Moreover, these high CCL3 levels predict a short time to first treatment, highlighting its pathogenic relevance of attracting T cells and myeloid cells to the TME [146].

Natural killer (NK) cells are increased in number, yet are functionally impaired in the peripheral blood of CLL patients [147,148]. The mechanisms by which NK cells are inhibited in CLL are poorly understood. NK cell suppression through cell-cell contact could occur through the expression of the tolerogenic MHC class I subtype HLA-G [149] and CD137L (4-1BB ligand) on CLL cells [150]. CLL cells can disrupt the lytic immune synapse formation between NK cells and their target cells [151]. NK cells appear to have an influence on disease progression, as the number of NK cells relative to the size of the CLL clone is related to time to first treatment [152].

Two BCR-related kinases, Bruton’s tyrosine kinase (BTK) and phosphoinositide 3-kinase δ (PI3Kδ), have been targeted with unparalleled clinical success (by the drugs ibrutinib and idelalisib, respectively). Syk, another kinase in the BCR signaling pathway, has also been investigated as a druggable target in CLL. It has become apparent that the clinical efficacy of these drugs relies on more than just the abrogation of BCR-dependent survival and proliferation signals. In addition to its role in BCR signaling, BTK is also involved in the signaling of other receptors related to B-cell migration and adhesion, such as the CXCR4 and CXCR5 chemokine receptors and adhesion molecules (integrins) [153,154,155]. BTK inhibition with ibrutinib has elegantly been shown to reduce the surface expression of CXCR4, resulting in reduced anchoring of CLL cells in the TME of lymphoid organs and their subsequent release into the peripheral blood. This phenomenon explains the rapid escalation of lymphocytosis paralleled by early lymph node shrinkage at the start of ibrutinib treatment in CLL patients. These circulating CLL cells fail to upregulate CXCR4, preventing them from re-entering the tissue niches. This effectively deprives them of survival signals, which eventually causes cell death [156]. Furthermore, ibrutinib treatment leads to an immediate downregulation of a plethora of chemokines in the plasma, mainly through alteration of CLL cells and TME cells in the lymph nodes [157]. The immunomodulatory effects of BCR signaling inhibitors has recently been reviewed by Maharaj et al. [158]. Interestingly, IL-4 expression increases surface IgM expression and reduces CXCR4 and CXCR5 and seems to render CLL cells resistant to BCR-signaling inhibitors ibrutinib and idelalisib [159]. In retrospect, the downmodulation of CXCR4 and CXCR5 by IL-4 might explain the lymphocytosis that was observed in an early clinical trial testing IL-4 therapy in CLL patients [160].

### 2.4. Classical Hodgkin Lymphoma

Classical Hodgkin lymphoma exemplifies an extreme tumor composition pattern, labelled ‘recruitment’ [3]. Only a small minority of cells in the tumor (approximately 1%) are the malignant Hodgkin Reed-Sternberg (HRS) cells, which are embedded in an inflammatory environment of immune cells making the tissue dissimilar to healthy lymph nodes. HRS cells most likely derive from GC B cells, but have a heavily reprogrammed gene expression profile with a loss of most of the typical B-cell genes and cell surface molecules, though they do express CD30 almost uniformly. While driving mutations and major transforming events are still poorly understood, recurrent genetic aberrations in constituents of the JAK/STAT and NFκB signaling pathways resulting in increased activity are common features [161].

Among the immune cells that are recruited to the microenvironment by HRS cells, T cells are the most frequent. CD4^+^ T cells are attracted through the secretion of CCL5 (also known as RANTES), CCL17 (also known as TARC), CCL20 and CCL22 [162,163,164]. These CD4^+^ T cells are predominantly of the Treg and T_H_2 subtype [165,166,167]. Compared to reactive lymph nodes, cHL lymph nodes feature an expansion of active PD-1^−^ Tregs and exhausted PD-1^+^ T_H_1-polarized effector T cells, collectively contributing to immunosuppression [168].

The secretion of IL-5, CCL5 and CCL28 by HRS cells attracts eosinophils, CCL5 also recruits mast cells and HRS-secreted IL-8 recruits neutrophils [162]. HRS cells also support their own survival by secreting CCL5 [164].

The survival and proliferation signals that HRS cells get from the microenvironment are induced by CD40 ligation and IL-3 production by CD40L-expressing CD4^+^ T cells, activation of CD30 by CD30L-expressing eosinophils and mast cells, and BCMA activation by APRIL-expressing neutrophils [161,169].

**Figure 1:**
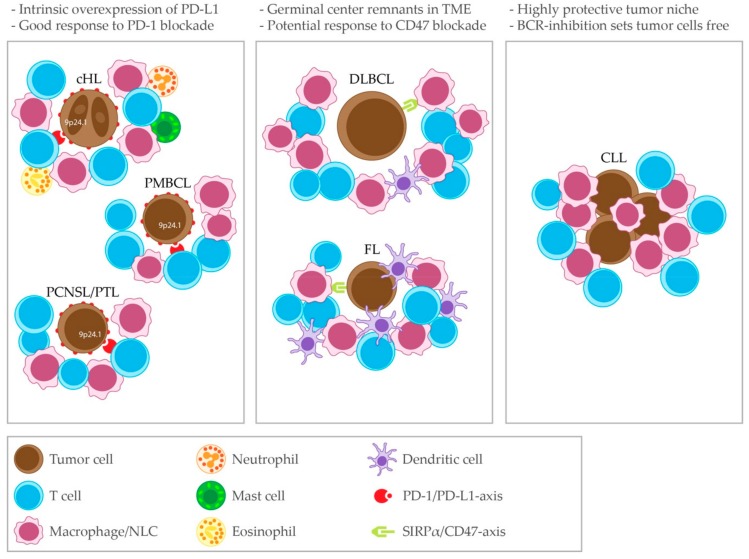
Common immune microenvironment patterns in lymphomas of B-cell origin. Left panel: cHL, PMBCL and PCNSL/PTL share the feature of genetic aberrations in the 9p24.1 locus leading to an intrinsic overexpression of PD-L1, which is associated with a good clinical response to PD-1 blockade. Middle panel: DLBCL and FL show remnants of the germinal center in their immune microenvironment and share the potential to clinically respond to CD47 blockade. Right panel: CLL has a distinct immune microenvironment which is markedly protective. BCR-inhibiting drugs release CLL cells from this niche. SIRPα = Signal regulatory protein α.

Multiple immune editing and immune evasion mechanisms are active in cHL. HRS cells can drive the differentiation of CD4^+^ T cells towards Tregs [170]. Tregs are abundant in cHL affected lymph nodes and they are suppressive to CD8^+^ cytotoxic T cells [165,167]. Paradoxically, the presence of Tregs has been associated with superior patient survival [171,172,173]. HRS cells themselves express the T cell-suppressive molecules TGFβ, IL-10, PGE2 and galectin 1 [162,167,174,175]. HRS cells also express PD-L1 to suppress T cells [176]. Moreover, analogous to PMBCL, PCNSL and PTL, as illustrated in Figure 1, nearly all newly diagnosed cHL patients have alterations in the PD-L1 and PD-L2 loci (9p24.1), which is associated with constitutive PD-L1 expression. Advanced stage cHL patients have an increased frequency of 9p24.1 amplifications and patients with this genetic alteration have a shorter progression-free survival (PFS) [177]. Furthermore, most patients have a loss or reduced expression of β2M and MHC class I and reduced expression of CIITA and MHC class II [62,178]. In a cohort of newly diagnosed patients, reduced MHC class I expression was linked to a shorter PFS, while reduced MHC class II expression had no prognostic implications [179]. In a cohort of heavily pre-treated patients treated with the anti-PD1 antibody nivolumab, superior PFS was associated with a higher level of 9p24.1 alterations and high PD-L1 expression. Here, MHC class II expression by HRS cells was predictive for complete response (CR) (and PFS in a subgroup of patients). However, 92% of responders in this cohort where MHC class I negative, suggesting that the efficacy of PD-1 blockade in relapsed/refractory (R/R) cHL does not depend on CD8^+^ T-cell mediated cytotoxicity [180].

The expression of PD-1 and PD-L1 in the TME correlated to poor OS in previously untreated patients [181,182]. Global leukocyte expression of PD-1 and PD-L1 in the TME and PD-L1 expression on HRS cells intensified in biopsies from relapsed cHL patients, compared to matched biopsies from the initial diagnosis [183].

A relevant role in the pathogenesis of cHL is also played by TAMs. A gene-expression profiling study identified that TAMs are associated to treatment failure [184]. An increased infiltration with CD68^+^ macrophages, as analyzed by immunohistochemistry, correlated with a shorter PFS and more frequent relapse after autologous hematopoietic cell transplantation (auto-HCT) [184]. A meta-analysis including 22 studies revealed that a high proportion of TAMs in the TME is predictive for poor OS [185]. In addition to HRS cells, TAMs also express PD-L1. The majority of PD-L1 expression in the TME might even be represented by TAMs [186]. PD-L1^+^ TAMs form a surrounding niche around PD-L1^+^ HRS cells and have widespread contact with PD-1^+^ T cells. PD-L1^+^ HRS cells interact predominantly with CD4^+^ T cells, expanding the indicative evidence that CD4^+^ T cells might mediate the clinical response to PD-1 blockade in cHL [186].

### 2.5. Extranodal Marginal Zone Lymphoma of Mucosa-Associated Lymphoid Tissue (MALT)

This indolent type of lymphoma typically emerges in an environment of chronic inflammation, such as persistent infection or autoimmune disease, and therefore represents a rather heterogeneous group of diagnoses when it comes to pathogenesis. The most frequent subtype is *Helicobacter pylori*-associated MALT lymphoma of the stomach [187], for which *Helicobacter pylori* eradication is considered the first therapeutic approach, leading to lymphoma regression in >50% of the cases [188]. All major recurrent chromosomal translocations result in constitutive activation of the NFκB pathway, yet preferentially associate with different anatomical sites. The t(11;18)(q21;q21) translocation is the most common one. It is mainly found in gastric and pulmonary MALT lymphomas and is associated with resistance to *Helicobacter pylori* eradication [189]. Genetic alterations in TNFAIP3, PIM1, cMyc, P53 and Myd88 have also been described [190]. Similar to all other low-grade lymphomas, MALT lymphomas can transform into aggressive lymphomas.

Mucosa-associated lymphoid tissue lymphoma cells are notoriously dependent on survival signals from the microenvironment, illustrated by the fact that they are hard to grow in vitro without supportive T cells and stromal cells [191]. Early studies suggested that *Helicobacter pylori*-specific tumor infiltrating T cells support the growth of MALT lymphoma cells [192]. A gene expression profiling study comparing non-malignant MALT tissue with MALT lymphoma tissue revealed an overexpression of CD1c, CD40, CD83, CD86, CD122 and HLA-D in the latter, suggesting the involvement of APCs and possibly T cells in this disease [193]. Infiltrating T cells are mostly Tregs and T_H_2 cells [194], which are presumably attracted by tumor-secreted CCL17 and CCL22 and offer support via CD40-CD40L interaction [195]. Furthermore, tumor cell-surrounding TAMs that sustain tumor growth via the secretion of APRIL have been described [196]. Further studies are required to comprehensively describe the composition of the TME in this lymphoma subtype.

### 2.6. Mantle Cell Lymphoma

Compared to more prevalent lymphomas, our knowledge of the TME content in MCL is limited. This lymphoma is characterized by small centrocytic lymphocytes in combination with a CD20^+^CD5^+^CCND1^+^ staining pattern. A blastoid variant also exists. The translocation t(11;14)(q13;q32) leading to cyclin D1 (CCND1) overexpression is a distinguishing characteristic of MCL [197]. Cyclin D1 could even serve as tumor-specific antigen for immunotherapy strategies [198]. Other established driver mutations are in ataxia-telangiectasia mutated (ATM) and TP53 [199]. MCL cells have a heavy survival dependency on stromal cells, especially in the context of resistance to chemotherapy [200]. The interactions with stromal cells are beyond the scope of this review.

MCL cells overexpress CCL4 and CCL5, chemokines that potentially attract Tregs to the TME [201]. The levels of infiltrating T cells, and specifically CD4^+^ T cells, are higher in indolent MCL than in more aggressive histological subtypes. A high CD4:CD8 ratio was an independent prognosticator for better OS [202]. Accordingly, a high absolute CD4^+^ T cell count and CD4:CD8 ratio in the peripheral blood correspond to good OS [203]. As reported by studies in bigger cohorts, infiltrating T cells do not express PD-1 and MCL cells do not express PD-L1 [36,37,204], although some smaller studies claim the opposite [205,206]. Furthermore, a low absolute NK cell count in the peripheral blood predicts poor OS [52]. It has also been suggested that TAMs play a role in in vitro and in vivo models of MCL [207,208]. A high absolute monocyte count in the peripheral blood of MCL patients has been associated with poor OS, though tumor-infiltrating TAMs did not predict prognosis [203,209]. MCL cells polarized monocytes towards CD163^+^ M2-like macrophages and received survival support from them ex vivo through the secretion of M-CSF and IL-10 and BTK inhibition blocked this process [210]. Accordingly, plasma concentrations of M-CSF and IL-10 are higher in MCL patients and peripheral blood monocyte-expressed CD163 is decreased in MCL patients that responded to combination treatment of ibrutinib and an anti-CD20 antibody [210]. Interestingly, ibrutinib enhanced the efficacy of an anti-PD-L1 antibody that was associated with an improved tumor-specific T-cell response in an ibrutinib-insensitive lymphoma mouse model [211]. Furthermore, BCR-signaling inhibitors (including ibrutinib) were shown to upregulate PD-L1 by MCL cells [206]. These studies justify further investigation into the combination of ibrutinib and an anti-PD-L1 antibody for MCL treatment.

## 3. Clinical Efficacy of Drugs That Target the Immune Tumor Microenvironment

The increasing knowledge of the TME composition and of the complex interactions between tumor cells and surrounding immune cells has triggered the development of drugs that target these mechanisms. Approaches aiming at unleashing an effective anti-tumor immune response by improving T-cell and macrophage functions have been investigated predominantly. ICB, especially against PD-1, has been very successful in certain lymphoma types. Table 2 provides a summary of the results of the published clinical trials with therapeutics targeting the TME.

### 3.1. Diffuse Large B-Cell Lymphoma

Ipilimumab, an anti-CTLA-1 antibody, has been tested in a phase 1 study in 18 R/R non-Hodgkin B-cell lymphoma patients. Of these, 3 had DLBCL and 1 achieved CR without relevant toxicity [212].

Nivolumab, an anti-PD-1 antibody, has been studied in a phase I trial in R/R B-cell lymphoma, T-cell lymphoma, and multiple myeloma. Of the 81 patients accrued, 11 had DLBCL. CR and partial response (PR) were achieved in 2 and 2 of these patients, respectively (overall response rate (ORR) 36%). No relevant toxicity was observed [213]. However, results from a subsequent phase II study in 121 DLBCL patients were disappointing, with an ORR of 10% in the auto-HCT-failed group and 3% in the auto-HCT-ineligible group, respectively. Median duration of response (DOR) was 11 and 8 months for these two subgroups, respectively [214]. On the other hand, in a small case series of 4 patients with R/R primary central nervous system (CNS) lymphoma (PCNSL) and 1 patient with a CNS relapse of primary testicular lymphoma (PTL), nivolumab treatment achieved an impressive ORR of 100% (4 CR and 1 PR) [51]. A phase 2 clinical trial using nivolumab treatment for patients with R/R PCNSL and PTL has been initiated (NCT02857426).

A phase 1/2a study assessing the safety and efficacy of the combination of ibrutinib and nivolumab in 45 R/R DLBCL patients (among other diagnoses) showed that this combination had an efficacy comparable to ibrutinib monotherapy with an ORR of 36% in DLBCL patients [215].

Pembrolizumab, another anti-PD-1 antibody, has been tested in R/R PMBCL patients. Seven out of 17 patients (41%) showed objective responses and 13 out of 16 patients (81%) with available imaging had a shrinkage of lesions in an interim analysis [216]. Reports of the use of pembrolizumab in general DLBCL patients are scarce. A single case report describes that pembrolizumab in combination with lenalidomide can lead to remission in refractory double-hit lymphoma [217]. Multiple trials that assess PD-1 blockade in combination with several other treatment modalities are currently ongoing. Among these, a combination with an anti-CD20 antibody is currently being tested. Pidilizumab, a proclaimed anti-PD-1 antibody, has been tested in patients directly post-auto-HCT. Of the 35 patients that had measurable disease after auto-HCT, 34% reached a CR and an additional 17% reached a PR (ORR 51%) [218].

Finally, CD47 blockade with Hu5FG-G4, which is expected to enable tumor cell phagocytosis, has shown promising clinical responses in a phase 1b dose escalation trial combined with rituximab. It included 15 DLBCL patients, of whom 33% achieved a CR and 7% a PR (ORR 40%). The most prominent (on-target) serious adverse event was anemia, which was rarely dose-limiting [219].

### 3.2. Follicular Lymphoma

In a pilot study including patients with different advanced malignancies who had been previously immunized with an anti-cancer vaccine and subsequently treated with ipilimumab, 2 FL patients achieved a PR and SD, respectively [220]. In some of the trials mentioned in the DLBCL section, FL patients were also treated. The clinical efficacy of ipilimumab was assessed in 14 patients with FL; of these, only one patient achieved a PR which lasted 19 months [212]. The efficacy of nivolumab, alone or in combination with ibrutinib, was also assessed. As single drug, ibrutinib achieved an ORR of 40% (10% CR) in 10 R/R patients [213]. The response rates of nivolumab achieved in combination with ibrutinib were similar [215]. In another trial, 32 R/R FL patients with rituximab-sensitive disease were treated with the combination of pembrolizumab and rituximab and a high CR rate was achieved (50%) [221].

### 3.3. Chronic Lymphocytic Leukemia

In CLL, the efficacy of PD-1 blockade has been tested in 2 different trials. Nivolumab has been tested in combination with ibrutinib in 36 patients with R/R CLL and 20 patients with Richter transformation (RT) and the ORRs were 61% and 65%, respectively, with no CR in the CLL group and 2 CRs in the RT group [215]. Also in the R/R setting, pembrolizumab has been tested in 16 CLL patients and 9 RT patients, of which 60% had previously been exposed to ibrutinib. In this study, no clinical responses were observed in CLL patients, while 4 out 9 RT patients achieved responses (2 CR). Interestingly, only patients who had progressed after ibrutinib responded and increased expression of PD-L1 and an increased trend of expression of PD-1 in pre-treatment tumor specimens from responding patients was observed compared to non-responding ones [222]. Three clinical trials are ongoing evaluating the combination of pembrolizumab either with the PI3Kδ inhibitor idelalisib or ibrutinib (NCT02332980); with the anti-CD20 antibody ublituximab (TG-1101) and the PI3Kδ inhibitor umbralisib (NCT02535286); and with fludarabine and ibrutinib (NCT03204188). Moreover, the combination of the anti-PD-L1 antibody atezolizumab with obinutuzumab and ibrutinib is also being tested (NCT02846623).

### 3.4. Classical Hodgkin Lymphoma

As mentioned above, there is convincing evidence that cHL has a genetically determined vulnerability to PD-1 blockade, since the genes encoding the PD-1 ligands, *PDL1* and *PDL2*, are key targets of chromosome 9p24.1 amplification, a recurrent genetic abnormality in cHL [46]. After an initial phase I trial showed promising activity in a group of 23 R/R cHL patients [223], the efficacy of nivolumab was tested in the post-auto-HCT setting in the phase II trial Checkmate-205. In this study, 243 patients were accrued into cohorts by treatment history: brentuximab vedotin (BV)-naive (cohort A), BV received after auto-HCT (cohort B), and BV received before and/or after auto-HCT (cohort C). Clinical benefit was observed in all different patient populations with ORRs ranging from 65% to 73%. The duration of the response increased with increasing depth of the response. However, OS was similar across response groups and continued benefit was observed beyond traditionally-defined disease progression [224]. This has led to proposed updates of the conventional response criteria when it comes to studies evaluating ICB [225]. In this trial, no increased incidence of acute graft-versus-host disease (GVHD) and transplant-related mortality (TRM) was reported in patients who subsequently underwent allogeneic hematopoietic cell transplantation (allo-HCT), as is reported elsewhere [226]. Larger studies are needed to evaluate whether PD-1 blockade can increase the risk of post-transplant toxicity. Nivolumab has also been tested in combination with BV, in a phase I-II trial enrolling patients with R/R cHL, with different dosing strategies (staggered vs concurrent). Patients in parts 1 and 2 received up to four 21-day cycles of staggered dosing (day 1: BV 1.8 mg/kg; day 8: nivolumab 3 mg/kg in cycle 1) and concurrent dosing thereafter. The observed ORR in the 61 evaluable patients was 82% (CR 61%) [227], with incidence and severity of adverse events similar to those reported for nivolumab and BV administered individually, with the exception of the incidence of infusion-related reactions, which was higher for unclear reasons. Patients in part 3 (n = 30) received up to four 21-day cycles of concurrent BV and nivolumab on day 1 and a 93% ORR (80% CR) was observed [228]. More than 80% of the treated patients proceeded directly to auto-HCT, confirming the efficacy of this combination as salvage therapy prior to auto-HCT.

Similarly, the proof-of-concept of pembrolizumab efficacy came from a phase I trial in 31 R/R cHL patients in which an ORR of 65% (16% CR) was achieved [229]. These results were subsequently confirmed in the phase II trial Keynote-087, in which three cohorts of R/R cHL patients were treated with single-agent pembrolizumab. The cohorts were defined based on disease progression after (1) auto-HCT and subsequent BV; (2) salvage chemotherapy including BV and (3) auto-HCT but had not received BV, and clinical outcome was similar to nivolumab in the same settings [230].

In the past years, anti-PD-1 monotherapy has represented a major advance in the treatment of patients with R/R cHL and both nivolumab and pembrolizumab were approved by FDA for the treatment of these patients, in slightly different settings (nivolumab in patients who relapsed after auto-HCT and post-transplantation BV; pembrolizumab in refractory patients or who have relapsed after ≥3 prior lines of therapy). However, despite the high ORRs, the CR rate is low. In an attempt to improve cure rates, PD-1 blockade was tested as consolidation after auto-HCT in 30 patients and the primary endpoint of improvement of 18 months-PFS from 60% to 80% was met. Toxicity was manageable, similar to what was reported in previous clinical trials [231]. Since the curative potential of PD-1 blockade as monotherapy appears to be low, new treatment combinations are worth investigating, including the combination with standard treatments (chemo/radiotherapy), with blockade of other immune checkpoints (CTLA-4, LAG-3, 4-1-BB) or with other drugs indirectly modulating the immune system (iMiDs, HDAC inhibitors, BTK-inhibitors, PI3K-inhibitors).

Finally, beyond the R/R disease, PD-1 blockade could also have a role in other disease settings, such as front-line, with the aim to eliminate radiotherapy in the early stages, to improve cure rates in advanced-stage (stage III–IV) disease, or for elderly patients, ineligible for chemotherapy. Indeed, a phase 2 trial (NCT03712202) is presently ongoing to assess whether the combination of nivolumab and BV can be incorporated in a radiation-free management of patients with early-stage cHL. Moreover, the combination of nivolumab with doxorubicin, vinblastine, dacarbazine (AVD), for example, is being compared with the combination of BV and AVD in patients with newly diagnosed advanced-stage cHL. The planned accrual in this trial (S1826; NCT03907488) is 470 patients in each arm. Finally, the combination of nivolumab and BV is being tested as primary treatment in patients over 60 years of age (NCT02758717).

### 3.5. Extranodal Marginal Zone Lymphoma of Mucosa-Associated Lymphoid Tissue

Very good CR rates are achieved with available standard treatment for this lymphoma subtype. Arguably due to this excellent response to first-line treatment and the insufficient knowledge of the TME in MALT lymphoma, drugs that directly target the immune microenvironment have not been tested in this subtype.

### 3.6. Mantle Cell Lymphoma

The rare MCL patients (n = 5) that were included in phase I clinical trials testing ICB drugs lacked objective responses [212,213], except for one patient with a PR after ipilimumab treatment post allo-HCT [232]. More pre-clinical studies are warranted to identify therapeutic strategies that could successfully target the immune microenvironment of this relentless subtype of NHL.

## 4. Conclusions and Perspectives

The outcome of treatment with drugs targeting the immune cells in the TME has been variable among the different B-cell lymphoma subtypes, ranging from very poor, such as in CLL, to good, such as in cHL, PMBCL and PCNSL, in which very high ORRs were achieved in the R/R setting. Intrinsic overexpression of PD-L1 is the presumed basis for the high ORRs in these diagnoses. Accordingly, cHL is the disease that shows better clinical response to PD-1 inhibitors than any other malignancy [233]. Translational research studies on the TME in cHL have led to the implementation of a new therapeutic strategy in a subgroup of patients which lacked treatment options, i.e., patients relapsing after auto-HSC. Indeed, the introduction of ICB therapy in cHL has been a story of success, bridging the gap between translational research and an unmet clinical need. This has paved the way for the investigation of PD-1 blockade in new clinical settings, such as before auto-HCT or as consolidation after auto-HCT.

The response rates to PD-1 blockade in R/R DLBCL have been disappointing, as is the case in FL. However, clinical responses were observed in 100% of CLL patients with RT who had progressed on ibrutinib, once more highlighting the importance of assessing drug efficacy in subgroups of patients with specific clinical characteristics.

Therapeutic blockade of CD47, expressed on lymphoma cells and inhibiting tumor cell phagocytosis by macrophages, showed promising response rates in combination with rituximab in both DLBCL and FL.

Taken together, these findings prompt further investigation into therapeutics that target the TME in lymphoma patients.

## Figures and Tables

**Table 1:** Key pathological features of lymphomas of B-cell origin.

Diagnosis	Immuno-Phenotype	Characteristic Chromosomal Aberrations	Typical Neoplastic Changes	Immune TME Composition
DLBCL	CD20^+^CD5^−^ CD23^−^	-	-	NK cells DCs macrophages T cells
CLL	CD20^+^CD5^+^ CD23^+^	del(11q) del(13q), del(17p), trisomy 12	-	Macrophages T cells
FL	CD20^+^CD10^+^ BCL2^+^BCL6^+^	t(14;18)(q32;p21)	*BCL2* overexpression	FDCs T cells (TFH) macrophages normal B cells
cHL	CD30^+^CD15^+^			T cells macrophages eosinophils mast cells neutrophils normal B cells
MALT	CD20^+^CD5^−^ CD10^−^			T cells FDCs macrophages normal B cells
MCL	CD20^+^CD5^+^ CD10^−^CD23^−^ CCND1^+^	t(11;14)(q13;q32)	*CCND1* overexpression	T cells macrophages
PMBCL	CD20^+^CD3^+^sIg^−^	-	-	T cells macrophages
SMZL	CD20^+^CD5^−^ CD10^-^CD23^-^			T cells normal B cells FDCs macrophages
BL	CD20^+^CD10^+^ BCL2^−^BCL6^+^	t(8;14)(q24;q32)	*MYC* overexpression	macrophages
NMZL	CD20^+^CD5^−^ CD10^−^CD23^-^	-	-	FDCs macrophages T cells normal B cells
LPL	CD20^+^CD10^−^ CD23^−^	-	-	T cells mast cells macrophages
NLPHL	CD20^+^CD30^−^ CD15^−^BCL6^+^			normal B cells FDCs macrophages T cells
THRLBCL	CD20^+^EMA^+^ BCL2^+^BCL6^+^	-	-	T cells macrophages

DLBCL = diffuse large B-cell lymphoma; CLL = chronic lymphocytic leukemia; FL = follicular lymphoma; cHL = classical Hodgkin lymphoma; MALT = extranodal marginal zone lymphoma of mucosa associated lymphoid tissue; MCL = mantle cell lymphoma; PMBCL = primary mediastinal large B-cell lymphoma; SMZL = splenic marginal zone lymphoma; BL = Burkitt lymphoma; NMZL = nodal marginal zone lymphoma; LPL = lymphoplasmacytic lymphoma; NLPHL = nodular lymphocyte predominant Hodgkin lymphoma; THRLBCL = T-cell/histiocyte rich large B-cell lymphoma; sIg = surface Immunoglobulin; NK = natural killer; DC = dendritic cell; TFH = follicular helper T cells; FDC = follicular dendritic cell.

**Table 2:** Published clinical trials with therapeutics targeting the immune tumor microenvironment.

Diagnosis	Therapeutics	Molecular Target	Target Cells	Trial Phase	Trial Name (Clinicaltrials.Gov Identifier)	Patients (N)	Clinical Setting	Clinical Response	Clinical Outcome	Reference
DLBCL	Ipilimumab	CTLA-1	T cells	I	(NCT00089076)	3	R/R	ORR 33% (33% CR)	TTP 31+ mo	Ansell, Hurvitz et al. 2009
	Nivolumab	PD-1	T cells	Ib	(NCT01592370)	11	R/R	ORR 36% (18% CR)	mPFS 7 wks	Lesokhin, Ansell et al. 2016
	Nivolumab	PD-1	T cells	II	(NCT02038933)	121				Ansell, Minnema et al. 2019
						87	auto-HCT-failed	ORR 10% (3% CR)	mTTP 11 mo	
						34	auto-HCT-ineligible	ORR 3% (0% CR)	mTTP 8 mo	
	Nivolumab + ibrutinib	PD-1 + BTK	T cells + B cells	I–IIa	(NCT02329847)	45	R/R	ORR 36% (16% CR)	mPFS 2.1 mo	Younes, Brody et al. 2019
	Pidilizumab	PD-1	T cells	Ib	(NCT00532259)	66	post- auto-HCT		16-mo PFS 0.72	Armand, Nagler et al. 2013
						35	with measurable disease post-auto-HCT	ORR 51% (CR 34%)		
	Hu5F9-G4 + rituximab	CD47 + CD20	Tumor cells	Ib	(NCT02953509)	15	R/R	ORR 40% (33% CR)	mDOR n.r. at 6.2 mo	Advani, Flinn et al. 2018
PCNSL	Nivolumab	PD-1	T cells	Ib	n/a	5 (1 PTL with CNS engagement)	R/R	ORR 100% (80% CR)	PFS 13+ -17+ mo	Nayak, Iwamoto et al. 2017
PMBCL	Pembrolizumab	PD-1	T cells	Ib	KEYNOTE-013 (NCT01953692)	17	R/R	ORR 41% (12% CR)	DOR 2.3+ - 22.5+ mo	Zinzani, Ribrag et al. 2017
FL	Ipilimumab	CTLA-1	T cells	I	(NCT00089076)	14	R/R	ORR 7% (0% CR)	TTP 19 mo	Ansell, Hurvitz et al. 2009
	Nivolumab	PD-1	T cells	Ib	(NCT01592370)	10	R/R	ORR 40% (10% CR)	N/R	Lesokhin, Ansell et al. 2016
	Nivolumab + ibrutinib	PD-1 + BTK	T cells + B cells	I–IIa	(NCT02329847)	40	R/R	ORR 33% (10% CR)	mPFS 9.1 mo	Younes, Brody et al. 2019
	Pembrolizumab + rituximab	PD-1 + CD20	T cells + B cells	II	n/a	32	R/R	ORR 67% (50% CR)	mPFS 11.4 mo	Nastoupil L, et al. 2017
	Hu5F9-G4 + rituximab	CD47 + CD20	Tumor cells	Ib	(NCT02953509)	7	R/R	ORR 71% (43% CR)	mDOR n.r. at 8.1 mo	Advani, Flinn et al. 2018
CLL	Nivolumab + ibrutinib	PD-1 + BTK	T cells + B cells	I–IIa	(NCT02329847)	36	R/R	ORR 61% (0% CR)	mPFS N/A	Younes, Brody et al. 2019
	Pembrolizumab	PD-1	T cells	II	(NCT02332980)	16	R/R	ORR 0%	mPFS 2.4 mo	Ding, LaPlant et al. 2017
						9	prior ibrutinib	ORR 0%		
RT	Nivolumab + ibrutinib	PD-1 + BTK	T cells + B cells	I–IIa	(NCT02329847)	20	R/R	ORR 65% (10% CR)	mPFS 5 mo	Younes, Brody et al. 2019
	Pembrolizumab	PD-1	T cells	II	(NCT02332980)	9	R/R	ORR 44% (11% CR)	mPFS 5.4 mo	Ding, LaPlant et al. 2017
						6	prior ibrutinib	ORR 100%		
cHL	Nivolumab	PD-1	T cells	I	(NCT01592370)	23	R/R	ORR 87% (CR 17%)	6mo PFS 86%	Ansell, Lesokhin et al. 2015
	Nivolumab	PD-1	T cells	II	CheckMate 205 (NCT02181738)	243	R/R			Armand, Engert et al. 2018
						63 (cohort A)	failed auto-HCT, BV-naïve	ORR 65% (29% CR)	mPFS 18.3 mo	
						80 (cohort B)	failed auto-HCT and BV after	ORR 68% (13% CR)	mPFS 14.7 mo	
						100 (cohort C)	failed auto-HCT, BV before and/or after auto-HCT	ORR 73% (12% CR)	mPFS 11.9 mo	
	Nivolumab + Brentuximab Vedotin	PD-1 + CD30	T cells + HRS cells	I–II	(NCT02572167)		R/R			Herrera, Moskowitz et al. 2018
						60 (groups 1–2)		ORR 82% (61% CR)	9-mo PFS* 86% 15-mo PFS* 82%	
						30 (group 3)		ORR 93% (80% CR)	9-mo PFS* 88%	
	Pembrolizumab	PD-1	T cells	I	KEYNOTE-013 (NCT01953692)	31	R/R, prior BV 71% prior auto-HSC	ORR 65% (16% CR)	6-mo PFS 69% 1-yr PFS 46%	Armand, Shipp et al. 2016
	Pembrolizumab	PD-1	T cells	II	KEYNOTE-087 (NCT02453594)		R/R			Chen, Zinzani et al. 2017
						69 (cohort 1)	failed auto-HCT and BV	ORR 74% (22% CR)		
						81 (cohort 2)	failed salvage incl. BV	ORR 64% (25% CR)		
						60 (cohort 3)	failed auto-HCT, no BV	ORR 70% (20% CR)		
	Pembrolizumab	PD-1	T cells	II	(NCT02362997)	30	consolidation after auto-HCT		18-mo PFS 82% 18-mo OS 100%	Armand, Chen et al. 2019

DLBCL = diffuse large B cell lymphoma; PCNSL = primary central nervous system (CNS) lymphoma; PMBCL = primary mediastinal large B-cell lymphoma; FL = follicular lymphoma; cHL = classical Hodgkin lymphoma; PTL = primary testicular lymphoma; RT = Richter transformation; R/R = relapsed/refractory; ORR = overall response rate; CR = complete response; mTTP = median time to progression; mDOR = median duration of response; N/A = not assessable; N/R = not reported; n.r. = not reached; mPFS = median progression-free survival; mo = months; wks = weeks; auto-HCT = autologous stem cell transplantation; HRS = Hodgkin Reed-Sternberg; BTK = Bruton’s tyrosine kinase. * estimated.

## References

[1] Swerdlow S.H., Campo E., Harris N.L., Jaffe E.S., Pileri S.A., Stein H., Thiele J. (2008). WHO Classification of Tumours of Haematopoietic and Lymphoid Tissues.

[2] World Cancer Research Fund International (WCRF). https://www.wcrf.org/dietandcancer/cancer-trends/worldwide-cancer-data.

[3] Scott D.W., Gascoyne R.D. (2014). The tumour microenvironment in B cell lymphomas. Nat. Rev. Cancer.

[4] Jares P., Colomer D., Campo E. (2012). Molecular pathogenesis of mantle cell lymphoma. J. Clin. Investig..

[5] Chiorazzi N., Ferrarini M. (2011). Cellular origin (s) of chronic lymphocytic leukemia: Cautionary notes and additional considerations and possibilities. Blood.

[6] Hanahan D., Weinberg R.A. (2011). Hallmarks of cancer: The next generation. Cell.

[7] Keir M.E., Butte M.J., Freeman G.J., Sharpe A.H. (2008). PD-1 and its ligands in tolerance and immunity. Annu. Rev. Immunol..

[8] Dong H., Strome S.E., Salomao D.R., Tamura H., Hirano F., Flies D.B., Roche P.C., Lu J., Zhu G., Tamada K. (2002). Tumor-associated B7-H1 promotes T-cell apoptosis: A potential mechanism of immune evasion. Nat. Med..

[9] Curiel T.J., Wei S., Dong H., Alvarez X., Cheng P., Mottram P., Krzysiek R., Knutson K.L., Daniel B., Zimmermann M.C. (2003). Blockade of B7-H1 improves myeloid dendritic cell-mediated antitumor immunity. Nat. Med..

[10] Chihara D., Ito H., Matsuda T., Shibata A., Katsumi A., Nakamura S., Tomotaka S., Morton L.M., Weisenburger D.D., Matsuo K. (2014). Differences in incidence and trends of haematological malignancies in Japan and the United States. Br. J. Haematol..

[11] Xie Y., Pittaluga S., Jaffe E.S. (2015). The histological classification of diffuse large B-cell lymphomas. Semin. Hematol..

[12] Alizadeh A.A., Eisen M.B., Davis R.E., Ma C., Lossos I.S., Rosenwald A., Boldrick J.C., Sabet H., Tran T., Yu X. (2000). Distinct types of diffuse large B-cell lymphoma identified by gene expression profiling. Nature.

[13] Ciavarella S., Vegliante M.C., Fabbri M., De Summa S., Melle F., Motta G., De Iuliis V., Opinto G., Enjuanes A., Rega S. (2018). Dissection of DLBCL microenvironment provides a gene expression-based predictor of survival applicable to formalin-fixed paraffin-embedded tissue. Ann. Oncol..

[14] Kridel R., Steidl C., Gascoyne R.D. (2015). Tumor-associated macrophages in diffuse large B-cell lymphoma. Haematologica.

[15] Lin Y., Gustafson M.P., Bulur P.A., Gastineau D.A., Witzig T.E., Dietz A.B. (2011). Immunosuppressive CD14+HLA-DR(low)/-monocytes in B-cell non-Hodgkin lymphoma. Blood.

[16] Xiu B., Lin Y., Grote D.M., Ziesmer S.C., Gustafson M.P., Maas M.L., Zhang Z., Dietz A.B., Porrata L.F., Novak A.J. (2015). IL-10 induces the development of immunosuppressive CD14(+)HLA-DR(low/-) monocytes in B-cell non-Hodgkin lymphoma. Blood Cancer J..

[17] Chao M.P., Alizadeh A.A., Tang C., Myklebust J.H., Varghese B., Gill S., Jan M., Cha A.C., Chan C.K., Tan B.T. (2010). Anti-CD47 antibody synergizes with rituximab to promote phagocytosis and eradicate non-Hodgkin lymphoma. Cell.

[18] Chao M.P., Tang C., Pachynski R.K., Chin R., Majeti R., Weissman I.L. (2011). Extranodal dissemination of non-Hodgkin lymphoma requires CD47 and is inhibited by anti-CD47 antibody therapy. Blood.

[19] Coutinho R., Clear A.J., Mazzola E., Owen A., Greaves P., Wilson A., Matthews J., Lee A., Alvarez R., da Silva M.G. (2015). Revisiting the immune microenvironment of diffuse large B-cell lymphoma using a tissue microarray and immunohistochemistry: Robust semi-automated analysis reveals CD3 and FoxP3 as potential predictors of response to R-CHOP. Haematologica.

[20] Li L., Sun R., Miao Y., Tran T., Adams L., Roscoe N., Xu B., Manyam G.C., Tan X., Zhang H. (2019). PD-1/PD-L1 expression and interaction by automated quantitative immunofluorescent analysis show adverse prognostic impact in patients with diffuse large B-cell lymphoma having T-cell infiltration: A study from the International DLBCL Consortium Program. Mod. Pathol..

[21] Chen Z., Deng X., Ye Y., Gao L., Zhang W., Liu W., Zhao S. (2019). Novel risk stratification of de novo diffuse large B cell lymphoma based on tumour-infiltrating T lymphocytes evaluated by flow cytometry. Ann. Hematol..

[22] Xu Y., Kroft S.H., McKenna R.W., Aquino D.B. (2001). Prognostic significance of tumour-infiltrating T lymphocytes and T-cell subsets in de novo diffuse large B-cell lymphoma: A multiparameter flow cytometry study. Br. J. Haematol..

[23] Lippman S.M., Spier C.M., Miller T.P., Slymen D.J., Rybski J.A., Grogan T.M. (1990). Tumor-infiltrating T-lymphocytes in B-cell diffuse large cell lymphoma related to disease course. Mod. Pathol..

[24] Keane C., Gill D., Vari F., Cross D., Griffiths L., Gandhi M. (2013). CD4(+) tumor infiltrating lymphocytes are prognostic and independent of R-IPI in patients with DLBCL receiving R-CHOP chemo-immunotherapy. Am. J. Hematol..

[25] Ansell S.M., Stenson M., Habermann T.M., Jelinek D.F., Witzig T.E. (2001). Cd4+ T-cell immune response to large B-cell non-Hodgkin’s lymphoma predicts patient outcome. J. Clin. Oncol..

[26] Ahearne M.J., Bhuller K., Hew R., Ibrahim H., Naresh K., Wagner S.D. (2014). Expression of PD-1 (CD279) and FoxP3 in diffuse large B-cell lymphoma. Virchows Arch..

[27] Serag El-Dien M.M., Abdou A.G., Asaad N.Y., Abd El-Wahed M.M., Kora M. (2017). Intratumoral FOXP3+ Regulatory T Cells in Diffuse Large B-Cell Lymphoma. Appl. Immunohistochem. Mol. Morphol..

[28] Monti S., Savage K.J., Kutok J.L., Feuerhake F., Kurtin P., Mihm M., Wu B., Pasqualucci L., Neuberg D., Aguiar R.C. (2005). Molecular profiling of diffuse large B-cell lymphoma identifies robust subtypes including one characterized by host inflammatory response. Blood.

[29] Chang K.C., Huang G.C., Jones D., Lin Y.H. (2007). Distribution patterns of dendritic cells and T cells in diffuse large B-cell lymphomas correlate with prognoses. Clin. Cancer Res..

[30] Jeong J., Oh E.J., Yang W.I., Kim S.J., Yoon S.O. (2017). Implications of infiltrating immune cells within bone marrow of patients with diffuse large B-cell lymphoma. Hum. Pathol..

[31] Rydstrom K., Linderoth J., Nyman H., Ehinger M., Joost P., Bendahl P.O., Leppa S., Jerkeman M. (2010). CD40 is a potential marker of favorable prognosis in patients with diffuse large B-cell lymphoma treated with immunochemotherapy. Leuk. Lymphoma.

[32] Pericart S., Tosolini M., Gravelle P., Rossi C., Traverse-Glehen A., Amara N., Franchet C., Martin E., Bezombes C., Laurent G. (2018). Profiling Immune Escape in Hodgkin’s and Diffuse large B-Cell Lymphomas Using the Transcriptome and Immunostaining. Cancers.

[33] Andorsky D.J., Yamada R.E., Said J., Pinkus G.S., Betting D.J., Timmerman J.M. (2011). Programmed death ligand 1 is expressed by non-hodgkin lymphomas and inhibits the activity of tumor-associated T cells. Clin. Cancer Res..

[34] Chen B.J., Chapuy B., Ouyang J., Sun H.H., Roemer M.G., Xu M.L., Yu H., Fletcher C.D., Freeman G.J., Shipp M.A. (2013). PD-L1 expression is characteristic of a subset of aggressive B-cell lymphomas and virus-associated malignancies. Clin. Cancer Res..

[35] Keane C., Vari F., Hertzberg M., Cao K.A., Green M.R., Han E., Seymour J.F., Hicks R.J., Gill D., Crooks P. (2015). Ratios of T-cell immune effectors and checkpoint molecules as prognostic biomarkers in diffuse large B-cell lymphoma: A population-based study. Lancet Haematol..

[36] Vranic S., Ghosh N., Kimbrough J., Bilalovic N., Bender R., Arguello D., Veloso Y., Dizdarevic A., Gatalica Z. (2016). PD-L1 Status in Refractory Lymphomas. PLoS ONE.

[37] Menter T., Bodmer-Haecki A., Dirnhofer S., Tzankov A. (2016). Evaluation of the diagnostic and prognostic value of PDL1 expression in Hodgkin and B-cell lymphomas. Hum. Pathol..

[38] Georgiou K., Chen L., Berglund M., Ren W., de Miranda N.F., Lisboa S., Fangazio M., Zhu S., Hou Y., Wu K. (2016). Genetic basis of PD-L1 overexpression in diffuse large B-cell lymphomas. Blood.

[39] Kwon D., Kim S., Kim P.J., Go H., Nam S.J., Paik J.H., Kim Y.A., Kim T.M., Heo D.S., Kim C.W. (2016). Clinicopathological analysis of programmed cell death 1 and programmed cell death ligand 1 expression in the tumour microenvironments of diffuse large B cell lymphomas. Histopathology.

[40] Kiyasu J., Miyoshi H., Hirata A., Arakawa F., Ichikawa A., Niino D., Sugita Y., Yufu Y., Choi I., Abe Y. (2015). Expression of programmed cell death ligand 1 is associated with poor overall survival in patients with diffuse large B-cell lymphoma. Blood.

[41] Xing W., Dresser K., Zhang R., Evens A.M., Yu H., Woda B.A., Chen B.J. (2016). PD-L1 expression in EBV-negative diffuse large B-cell lymphoma: Clinicopathologic features and prognostic implications. Oncotarget.

[42] Rossille D., Gressier M., Damotte D., Maucort-Boulch D., Pangault C., Semana G., Le Gouill S., Haioun C., Tarte K., Lamy T. (2014). High level of soluble programmed cell death ligand 1 in blood impacts overall survival in aggressive diffuse large B-Cell lymphoma: Results from a French multicenter clinical trial. Leukemia.

[43] Meier C., Hoeller S., Bourgau C., Hirschmann P., Schwaller J., Went P., Pileri S.A., Reiter A., Dirnhofer S., Tzankov A. (2009). Recurrent numerical aberrations of JAK2 and deregulation of the JAK2-STAT cascade in lymphomas. Mod. Pathol..

[44] Twa D.D., Chan F.C., Ben-Neriah S., Woolcock B.W., Mottok A., Tan K.L., Slack G.W., Gunawardana J., Lim R.S., McPherson A.W. (2014). Genomic rearrangements involving programmed death ligands are recurrent in primary mediastinal large B-cell lymphoma. Blood.

[45] Bledsoe J.R., Redd R.A., Hasserjian R.P., Soumerai J.D., Nishino H.T., Boyer D.F., Ferry J.A., Zukerberg L.R., Harris N.L., Abramson J.S. (2016). The immunophenotypic spectrum of primary mediastinal large B-cell lymphoma reveals prognostic biomarkers associated with outcome. Am. J. Hematol..

[46] Green M.R., Monti S., Rodig S.J., Juszczynski P., Currie T., O’Donnell E., Chapuy B., Takeyama K., Neuberg D., Golub T.R. (2010). Integrative analysis reveals selective 9p24.1 amplification, increased PD-1 ligand expression, and further induction via JAK2 in nodular sclerosing Hodgkin lymphoma and primary mediastinal large B-cell lymphoma. Blood.

[47] Chang C., Lin C.H., Cheng A.L., Medeiros L.J., Chang K.C. (2015). Primary central nervous system diffuse large B-cell lymphoma has poorer immune cell infiltration and prognosis than its peripheral counterpart. Histopathology.

[48] Leivonen S.K., Pollari M., Bruck O., Pellinen T., Autio M., Karjalainen-Lindsberg M.L., Mannisto S., Kellokumpu-Lehtinen P.L., Kallioniemi O., Mustjoki S. (2019). T-cell inflamed tumor microenvironment predicts favorable prognosis in primary testicular lymphoma. Haematologica.

[49] Berghoff A.S., Ricken G., Widhalm G., Rajky O., Hainfellner J.A., Birner P., Raderer M., Preusser M. (2014). PD1 (CD279) and PD-L1 (CD274, B7H1) expression in primary central nervous system lymphomas (PCNSL). Clin. Neuropathol..

[50] Chapuy B., Roemer M.G., Stewart C., Tan Y., Abo R.P., Zhang L., Dunford A.J., Meredith D.M., Thorner A.R., Jordanova E.S. (2016). Targetable genetic features of primary testicular and primary central nervous system lymphomas. Blood.

[51] Nayak L., Iwamoto F.M., LaCasce A., Mukundan S., Roemer M.G.M., Chapuy B., Armand P., Rodig S.J., Shipp M.A. (2017). PD-1 blockade with nivolumab in relapsed/refractory primary central nervous system and testicular lymphoma. Blood.

[52] Zhou Y., Zha J., Lin Z., Fang Z., Zeng H., Zhao J., Luo Y., Li Z., Xu B. (2018). CD4+ T cell-mediated cytotoxicity is associated with MHC class II expression on malignant CD19+ B cells in diffuse large B cell lymphoma. Exp. Cell Res..

[53] Stopeck A.T., Gessner A., Miller T.P., Hersh E.M., Johnson C.S., Cui H., Frutiger Y., Grogan T.M. (2000). Loss of B7.2 (CD86) and intracellular adhesion molecule 1 (CD54) expression is associated with decreased tumor-infiltrating T lymphocytes in diffuse B-cell large-cell lymphoma. Clin. Cancer Res..

[54] Rimsza L.M., Roberts R.A., Miller T.P., Unger J.M., LeBlanc M., Braziel R.M., Weisenberger D.D., Chan W.C., Muller-Hermelink H.K., Jaffe E.S. (2004). Loss of MHC class II gene and protein expression in diffuse large B-cell lymphoma is related to decreased tumor immunosurveillance and poor patient survival regardless of other prognostic factors: A follow-up study from the Leukemia and Lymphoma Molecular Profiling Project. Blood.

[55] Rimsza L.M., Farinha P., Fuchs D.A., Masoudi H., Connors J.M., Gascoyne R.D. (2007). HLA-DR protein status predicts survival in patients with diffuse large B-cell lymphoma treated on the MACOP-B chemotherapy regimen. Leuk. Lymphoma.

[56] Jesionek-Kupnicka D., Bojo M., Prochorec-Sobieszek M., Szumera-Cieckiewicz A., Jablonska J., Kalinka-Warzocha E., Kordek R., Mlynarski W., Robak T., Warzocha K. (2016). HLA-G and MHC Class II Protein Expression in Diffuse Large B-Cell Lymphoma. Arch. Immunol. Ther. Exp..

[57] Riemersma S.A., Jordanova E.S., Schop R.F., Philippo K., Looijenga L.H., Schuuring E., Kluin P.M. (2000). Extensive genetic alterations of the HLA region, including homozygous deletions of HLA class II genes in B-cell lymphomas arising in immune-privileged sites. Blood.

[58] Rimsza L.M., Roberts R.A., Campo E., Grogan T.M., Bea S., Salaverria I., Zettl A., Rosenwald A., Ott G., Muller-Hermelink H.K. (2006). Loss of major histocompatibility class II expression in non-immune-privileged site diffuse large B-cell lymphoma is highly coordinated and not due to chromosomal deletions. Blood.

[59] Cycon K.A., Rimsza L.M., Murphy S.P. (2009). Alterations in CIITA constitute a common mechanism accounting for downregulation of MHC class II expression in diffuse large B-cell lymphoma (DLBCL). Exp. Hematol..

[60] Wilkinson S.T., Vanpatten K.A., Fernandez D.R., Brunhoeber P., Garsha K.E., Glinsmann-Gibson B.J., Grogan T.M., Teruya-Feldstein J., Rimsza L.M. (2012). Partial plasma cell differentiation as a mechanism of lost major histocompatibility complex class II expression in diffuse large B-cell lymphoma. Blood.

[61] Roberts R.A., Wright G., Rosenwald A.R., Jaramillo M.A., Grogan T.M., Miller T.P., Frutiger Y., Chan W.C., Gascoyne R.D., Ott G. (2006). Loss of major histocompatibility class II gene and protein expression in primary mediastinal large B-cell lymphoma is highly coordinated and related to poor patient survival. Blood.

[62] Steidl C., Shah S.P., Woolcock B.W., Rui L., Kawahara M., Farinha P., Johnson N.A., Zhao Y., Telenius A., Neriah S.B. (2011). MHC class II transactivator CIITA is a recurrent gene fusion partner in lymphoid cancers. Nature.

[63] Mottok A., Woolcock B., Chan F.C., Tong K.M., Chong L., Farinha P., Telenius A., Chavez E., Ramchandani S., Drake M. (2015). Genomic Alterations in CIITA Are Frequent in Primary Mediastinal Large B Cell Lymphoma and Are Associated with Diminished MHC Class II Expression. Cell Rep..

[64] Phipps-Yonas H., Cui H., Sebastiao N., Brunhoeber P.S., Haddock E., Deymier M.J., Klapper W., Lybarger L., Roe D.J., Hastings K.T. (2013). Low GILT Expression is Associated with Poor Patient Survival in Diffuse Large B-Cell Lymphoma. Front. Immunol..

[65] Brown P.J., Wong K.K., Felce S.L., Lyne L., Spearman H., Soilleux E.J., Pedersen L.M., Moller M.B., Green T.M., Gascoyne D.M. (2016). FOXP1 suppresses immune response signatures and MHC class II expression in activated B-cell-like diffuse large B-cell lymphomas. Leukemia.

[66] Challa-Malladi M., Lieu Y.K., Califano O., Holmes A.B., Bhagat G., Murty V.V., Dominguez-Sola D., Pasqualucci L., Dalla-Favera R. (2011). Combined genetic inactivation of beta2-Microglobulin and CD58 reveals frequent escape from immune recognition in diffuse large B cell lymphoma. Cancer Cell.

[67] Cycon K.A., Mulvaney K., Rimsza L.M., Persky D., Murphy S.P. (2013). Histone deacetylase inhibitors activate CIITA and MHC class II antigen expression in diffuse large B-cell lymphoma. Immunology.

[68] Jiang Y., Ortega-Molina A., Geng H., Ying H.Y., Hatzi K., Parsa S., McNally D., Wang L., Doane A.S., Agirre X. (2017). CREBBP Inactivation Promotes the Development of HDAC3-Dependent Lymphomas. Cancer Discov..

[69] Hashwah H., Schmid C.A., Kasser S., Bertram K., Stelling A., Manz M.G., Muller A. (2017). Inactivation of CREBBP expands the germinal center B cell compartment, down-regulates MHCII expression and promotes DLBCL growth. Proc. Natl. Acad. Sci. USA.

[70] Ennishi D., Takata K., Beguelin W., Duns G., Mottok A., Farinha P., Bashashati A., Saberi S., Boyle M., Meissner B. (2019). Molecular and Genetic Characterization of MHC Deficiency Identifies EZH2 as Therapeutic Target for Enhancing Immune Recognition. Cancer Discov..

[71] Fontan L., Qiao Q., Hatcher J.M., Casalena G., Us I., Teater M., Durant M., Du G., Xia M., Bilchuk N. (2018). Specific covalent inhibition of MALT1 paracaspase suppresses B cell lymphoma growth. J. Clin. Investig..

[72] Xia X., Zhou W., Guo C., Fu Z., Zhu L., Li P., Xu Y., Zheng L., Zhang H., Shan C. (2018). Glutaminolysis Mediated by MALT1 Protease Activity Facilitates PD-L1 Expression on ABC-DLBCL Cells and Contributes to Their Immune Evasion. Front. Oncol..

[73] Pascual M., Mena-Varas M., Robles E.F., Garcia-Barchino M.J., Panizo C., Hervas-Stubbs S., Alignani D., Sagardoy A., Martinez J.I., Bunting K.L. (2019). PD-1/PD-L1 immune checkpoint and p53 loss facilitate tumor progression in activated B cell diffuse large B-cell lymphomas. Blood.

[74] Kuppers R., Stevenson F.K. (2018). Critical influences on the pathogenesis of follicular lymphoma. Blood.

[75] Tweeddale M.E., Lim B., Jamal N., Robinson J., Zalcberg J., Lockwood G., Minden M.D., Messner H.A. (1987). The presence of clonogenic cells in high-grade malignant lymphoma: A prognostic factor. Blood.

[76] Ame-Thomas P., Maby-El Hajjami H., Monvoisin C., Jean R., Monnier D., Caulet-Maugendre S., Guillaudeux T., Lamy T., Fest T., Tarte K. (2007). Human mesenchymal stem cells isolated from bone marrow and lymphoid organs support tumor B-cell growth: Role of stromal cells in follicular lymphoma pathogenesis. Blood.

[77] Zhu D., McCarthy H., Ottensmeier C.H., Johnson P., Hamblin T.J., Stevenson F.K. (2002). Acquisition of potential N-glycosylation sites in the immunoglobulin variable region by somatic mutation is a distinctive feature of follicular lymphoma. Blood.

[78] Devan J., Janikova A., Mraz M. (2018). New concepts in follicular lymphoma biology: From BCL2 to epigenetic regulators and non-coding RNAs. Semin. Oncol..

[79] Raffeld M., Neckers L., Longo D.L., Cossman J. (1985). Spontaneous alteration of idiotype in a monoclonal B-cell lymphoma. Escape from detection by anti-idiotype. N. Engl. J. Med..

[80] Meeker T., Lowder J., Cleary M.L., Stewart S., Warnke R., Sklar J., Levy R. (1985). Emergence of idiotype variants during treatment of B-cell lymphoma with anti-idiotype antibodies. N. Engl. J. Med..

[81] Sachen K.L., Strohman M.J., Singletary J., Alizadeh A.A., Kattah N.H., Lossos C., Mellins E.D., Levy S., Levy R. (2012). Self-antigen recognition by follicular lymphoma B-cell receptors. Blood.

[82] Cha S.C., Qin H., Kannan S., Rawal S., Watkins L.S., Baio F.E., Wu W., Ong J., Wei J., Kwak B. (2013). Nonstereotyped lymphoma B cell receptors recognize vimentin as a shared autoantigen. J. Immunol..

[83] Coelho V., Krysov S., Ghaemmaghami A.M., Emara M., Potter K.N., Johnson P., Packham G., Martinez-Pomares L., Stevenson F.K. (2010). Glycosylation of surface Ig creates a functional bridge between human follicular lymphoma and microenvironmental lectins. Proc. Natl. Acad. Sci. USA.

[84] Green M.R., Kihira S., Liu C.L., Nair R.V., Salari R., Gentles A.J., Irish J., Stehr H., Vicente-Duenas C., Romero-Camarero I. (2015). Mutations in early follicular lymphoma progenitors are associated with suppressed antigen presentation. Proc. Natl. Acad. Sci. USA.

[85] Amin R., Mourcin F., Uhel F., Pangault C., Ruminy P., Dupre L., Guirriec M., Marchand T., Fest T., Lamy T. (2015). DC-SIGN-expressing macrophages trigger activation of mannosylated IgM B-cell receptor in follicular lymphoma. Blood.

[86] Calvo K.R., Dabir B., Kovach A., Devor C., Bandle R., Bond A., Shih J.H., Jaffe E.S. (2008). IL-4 protein expression and basal activation of Erk in vivo in follicular lymphoma. Blood.

[87] Pangault C., Ame-Thomas P., Ruminy P., Rossille D., Caron G., Baia M., De Vos J., Roussel M., Monvoisin C., Lamy T. (2010). Follicular lymphoma cell niche: Identification of a preeminent IL-4-dependent T(FH)-B cell axis. Leukemia.

[88] Pandey S., Mourcin F., Marchand T., Nayar S., Guirriec M., Pangault C., Monvoisin C., Ame-Thomas P., Guilloton F., Dulong J. (2017). IL-4/CXCL12 loop is a key regulator of lymphoid stroma function in follicular lymphoma. Blood.

[89] Victora G.D., Nussenzweig M.C. (2012). Germinal centers. Annu. Rev. Immunol..

[90] Caron G., Le Gallou S., Lamy T., Tarte K., Fest T. (2009). CXCR4 expression functionally discriminates centroblasts versus centrocytes within human germinal center B cells. J. Immunol..

[91] Epron G., Ame-Thomas P., Le Priol J., Pangault C., Dulong J., Lamy T., Fest T., Tarte K. (2012). Monocytes and T cells cooperate to favor normal and follicular lymphoma B-cell growth: Role of IL-15 and CD40L signaling. Leukemia.

[92] Ame-Thomas P., Tarte K. (2014). The yin and the yang of follicular lymphoma cell niches: Role of microenvironment heterogeneity and plasticity. Semin. Cancer Biol..

[93] Wahlin B.E., Sander B., Christensson B., Ostenstad B., Holte H., Brown P.D., Sundstrom C., Kimby E. (2012). Entourage: The immune microenvironment following follicular lymphoma. Blood Cancer J..

[94] Yang Z.Z., Grote D.M., Ziesmer S.C., Xiu B., Novak A.J., Ansell S.M. (2015). PD-1 expression defines two distinct T-cell sub-populations in follicular lymphoma that differentially impact patient survival. Blood Cancer J..

[95] Carreras J., Lopez-Guillermo A., Roncador G., Villamor N., Colomo L., Martinez A., Hamoudi R., Howat W.J., Montserrat E., Campo E. (2009). High numbers of tumor-infiltrating programmed cell death 1-positive regulatory lymphocytes are associated with improved overall survival in follicular lymphoma. J. Clin. Oncol..

[96] Wahlin B.E., Aggarwal M., Montes-Moreno S., Gonzalez L.F., Roncador G., Sanchez-Verde L., Christensson B., Sander B., Kimby E. (2010). A unifying microenvironment model in follicular lymphoma: Outcome is predicted by programmed death-1--positive, regulatory, cytotoxic, and helper T cells and macrophages. Clin. Cancer Res..

[97] Richendollar B.G., Pohlman B., Elson P., Hsi E.D. (2011). Follicular programmed death 1-positive lymphocytes in the tumor microenvironment are an independent prognostic factor in follicular lymphoma. Hum. Pathol..

[98] Yang Z.Z., Kim H.J., Villasboas J.C., Price-Troska T., Jalali S., Wu H., Luchtel R.A., Polley M.C., Novak A.J., Ansell S.M. (2019). Mass Cytometry Analysis Reveals that Specific Intratumoral CD4(+) T Cell Subsets Correlate with Patient Survival in Follicular Lymphoma. Cell Rep..

[99] Junlen H.R., Peterson S., Kimby E., Lockmer S., Linden O., Nilsson-Ehle H., Erlanson M., Hagberg H., Radlund A., Hagberg O. (2015). Follicular lymphoma in Sweden: Nationwide improved survival in the rituximab era, particularly in elderly women: A Swedish Lymphoma Registry study. Leukemia.

[100] Anastasia A., Rossi G. (2016). Novel Drugs in Follicular Lymphoma. Mediterr. J. Hematol. Infect. Dis..

[101] Cheah C.Y., Fowler N.H. (2018). Novel agents for relapsed and refractory follicular lymphoma. Best Pract. Res. Clin. Haematol..

[102] Wahlin B.E., Sundstrom C., Holte H., Hagberg H., Erlanson M., Nilsson-Ehle H., Linden O., Nordstrom M., Ostenstad B., Geisler C.H. (2011). T cells in tumors and blood predict outcome in follicular lymphoma treated with rituximab. Clin. Cancer Res..

[103] Herndon T.M., Chen S.S., Saba N.S., Valdez J., Emson C., Gatmaitan M., Tian X., Hughes T.E., Sun C., Arthur D.C. (2017). Direct in vivo evidence for increased proliferation of CLL cells in lymph nodes compared to bone marrow and peripheral blood. Leukemia.

[104] Burger J.A., Tsukada N., Burger M., Zvaifler N.J., Dell’Aquila M., Kipps T.J. (2000). Blood-derived nurse-like cells protect chronic lymphocytic leukemia B cells from spontaneous apoptosis through stromal cell-derived factor-1. Blood.

[105] Burkle A., Niedermeier M., Schmitt-Graff A., Wierda W.G., Keating M.J., Burger J.A. (2007). Overexpression of the CXCR5 chemokine receptor, and its ligand, CXCL13 in B-cell chronic lymphocytic leukemia. Blood.

[106] Jia L., Clear A., Liu F.T., Matthews J., Uddin N., McCarthy A., Hoxha E., Durance C., Iqbal S., Gribben J.G. (2014). Extracellular HMGB1 promotes differentiation of nurse-like cells in chronic lymphocytic leukemia. Blood.

[107] Polk A., Lu Y., Wang T., Seymour E., Bailey N.G., Singer J.W., Boonstra P.S., Lim M.S., Malek S., Wilcox R.A. (2016). Colony-Stimulating Factor-1 Receptor Is Required for Nurse-like Cell Survival in Chronic Lymphocytic Leukemia. Clin. Cancer Res..

[108] Boissard F., Fournie J.J., Laurent C., Poupot M., Ysebaert L. (2015). Nurse like cells: Chronic lymphocytic leukemia associated macrophages. Leuk. Lymphoma.

[109] Boissard F., Laurent C., Ramsay A.G., Quillet-Mary A., Fournie J.J., Poupot M., Ysebaert L. (2016). Nurse-like cells impact on disease progression in chronic lymphocytic leukemia. Blood Cancer J..

[110] Bhattacharya N., Diener S., Idler I.S., Rauen J., Habe S., Busch H., Habermann A., Zenz T., Dohner H., Stilgenbauer S. (2011). Nurse-like cells show deregulated expression of genes involved in immunocompetence. Br. J. Haematol..

[111] Kurtova A.V., Balakrishnan K., Chen R., Ding W., Schnabl S., Quiroga M.P., Sivina M., Wierda W.G., Estrov Z., Keating M.J. (2009). Diverse marrow stromal cells protect CLL cells from spontaneous and drug-induced apoptosis: Development of a reliable and reproducible system to assess stromal cell adhesion-mediated drug resistance. Blood.

[112] Deaglio S., Vaisitti T., Bergui L., Bonello L., Horenstein A.L., Tamagnone L., Boumsell L., Malavasi F. (2005). CD38 and CD100 lead a network of surface receptors relaying positive signals for B-CLL growth and survival. Blood.

[113] Nishio M., Endo T., Tsukada N., Ohata J., Kitada S., Reed J.C., Zvaifler N.J., Kipps T.J. (2005). Nurselike cells express BAFF and APRIL, which can promote survival of chronic lymphocytic leukemia cells via a paracrine pathway distinct from that of SDF-1alpha. Blood.

[114] Burger J.A., Quiroga M.P., Hartmann E., Burkle A., Wierda W.G., Keating M.J., Rosenwald A. (2009). High-level expression of the T-cell chemokines CCL3 and CCL4 by chronic lymphocytic leukemia B cells in nurselike cell cocultures and after BCR stimulation. Blood.

[115] Boissard F., Tosolini M., Ligat L., Quillet-Mary A., Lopez F., Fournie J.J., Ysebaert L., Poupot M. (2017). Nurse-like cells promote CLL survival through LFA-3/CD2 interactions. Oncotarget.

[116] Ten Hacken E., Burger J.A. (2014). Molecular pathways: Targeting the microenvironment in chronic lymphocytic leukemia--focus on the B-cell receptor. Clin. Cancer Res..

[117] Burger J.A., Burger M., Kipps T.J. (1999). Chronic lymphocytic leukemia B cells express functional CXCR4 chemokine receptors that mediate spontaneous migration beneath bone marrow stromal cells. Blood.

[118] Trentin L., Cabrelle A., Facco M., Carollo D., Miorin M., Tosoni A., Pizzo P., Binotto G., Nicolardi L., Zambello R. (2004). Homeostatic chemokines drive migration of malignant B cells in patients with non-Hodgkin lymphomas. Blood.

[119] Quiroga M.P., Balakrishnan K., Kurtova A.V., Sivina M., Keating M.J., Wierda W.G., Gandhi V., Burger J.A. (2009). B-cell antigen receptor signaling enhances chronic lymphocytic leukemia cell migration and survival: Specific targeting with a novel spleen tyrosine kinase inhibitor, R406. Blood.

[120] Panayiotidis P., Jones D., Ganeshaguru K., Foroni L., Hoffbrand A.V. (1996). Human bone marrow stromal cells prevent apoptosis and support the survival of chronic lymphocytic leukaemia cells in vitro. Br. J. Haematol..

[121] Lagneaux L., Delforge A., Bron D., De Bruyn C., Stryckmans P. (1998). Chronic lymphocytic leukemic B cells but not normal B cells are rescued from apoptosis by contact with normal bone marrow stromal cells. Blood.

[122] Mohle R., Failenschmid C., Bautz F., Kanz L. (1999). Overexpression of the chemokine receptor CXCR4 in B cell chronic lymphocytic leukemia is associated with increased functional response to stromal cell-derived factor-1 (SDF-1). Leukemia.

[123] Sivina M., Hartmann E., Vasyutina E., Boucas J.M., Breuer A., Keating M.J., Wierda W.G., Rosenwald A., Herling M., Burger J.A. (2012). Stromal cells modulate TCL1 expression, interacting AP-1 components and TCL1-targeting micro-RNAs in chronic lymphocytic leukemia. Leukemia.

[124] Ranheim E.A., Kipps T.J. (1993). Activated T cells induce expression of B7/BB1 on normal or leukemic B cells through a CD40-dependent signal. J. Exp. Med..

[125] Dazzi F., D’Andrea E., Biasi G., De Silvestro G., Gaidano G., Schena M., Tison T., Vianello F., Girolami A., Caligaris-Cappio F. (1995). Failure of B cells of chronic lymphocytic leukemia in presenting soluble and alloantigens. Clin. Immunol. Immunopathol..

[126] Molica S. (1994). Infections in chronic lymphocytic leukemia: Risk factors, and impact on survival, and treatment. Leuk. Lymphoma.

[127] Bartik M.M., Welker D., Kay N.E. (1998). Impairments in immune cell function in B cell chronic lymphocytic leukemia. Semin. Oncol..

[128] Kunicka J.E., Platsoucas C.D. (1988). Defective helper function of purified T4 cells and excessive suppressor activity of purified T8 cells in patients with B-cell chronic lymphocytic leukemia. T4 suppressor effector cells are present in certain patients. Blood.

[129] Rossi E., Matutes E., Morilla R., Owusu-Ankomah K., Heffernan A.M., Catovsky D. (1996). Zeta chain and CD28 are poorly expressed on T lymphocytes from chronic lymphocytic leukemia. Leukemia.

[130] Gorgun G., Holderried T.A., Zahrieh D., Neuberg D., Gribben J.G. (2005). Chronic lymphocytic leukemia cells induce changes in gene expression of CD4 and CD8 T cells. J. Clin. Investig..

[131] Pallasch C.P., Ulbrich S., Brinker R., Hallek M., Uger R.A., Wendtner C.M. (2009). Disruption of T cell suppression in chronic lymphocytic leukemia by CD200 blockade. Leuk. Res..

[132] Nunes C., Wong R., Mason M., Fegan C., Man S., Pepper C. (2012). Expansion of a CD8(+)PD-1(+) replicative senescence phenotype in early stage CLL patients is associated with inverted CD4:CD8 ratios and disease progression. Clin. Cancer Res..

[133] Brusa D., Serra S., Coscia M., Rossi D., D’Arena G., Laurenti L., Jaksic O., Fedele G., Inghirami G., Gaidano G. (2013). The PD-1/PD-L1 axis contributes to T-cell dysfunction in chronic lymphocytic leukemia. Haematologica.

[134] Palma M., Gentilcore G., Heimersson K., Mozaffari F., Nasman-Glaser B., Young E., Rosenquist R., Hansson L., Osterborg A., Mellstedt H. (2017). T cells in chronic lymphocytic leukemia display dysregulated expression of immune checkpoints and activation markers. Haematologica.

[135] Riches J.C., Davies J.K., McClanahan F., Fatah R., Iqbal S., Agrawal S., Ramsay A.G., Gribben J.G. (2013). T cells from CLL patients exhibit features of T-cell exhaustion but retain capacity for cytokine production. Blood.

[136] Ramsay A.G., Johnson A.J., Lee A.M., Gorgun G., Le Dieu R., Blum W., Byrd J.C., Gribben J.G. (2008). Chronic lymphocytic leukemia T cells show impaired immunological synapse formation that can be reversed with an immunomodulating drug. J. Clin. Investig..

[137] Ramsay A.G., Clear A.J., Fatah R., Gribben J.G. (2012). Multiple inhibitory ligands induce impaired T-cell immunologic synapse function in chronic lymphocytic leukemia that can be blocked with lenalidomide: Establishing a reversible immune evasion mechanism in human cancer. Blood.

[138] Ramsay A.G., Evans R., Kiaii S., Svensson L., Hogg N., Gribben J.G. (2013). Chronic lymphocytic leukemia cells induce defective LFA-1-directed T-cell motility by altering Rho GTPase signaling that is reversible with lenalidomide. Blood.

[139] Beyer M., Kochanek M., Darabi K., Popov A., Jensen M., Endl E., Knolle P.A., Thomas R.K., von Bergwelt-Baildon M., Debey S. (2005). Reduced frequencies and suppressive function of CD4+CD25hi regulatory T cells in patients with chronic lymphocytic leukemia after therapy with fludarabine. Blood.

[140] Pizzolo G., Chilosi M., Ambrosetti A., Semenzato G., Fiore-Donati L., Perona G. (1983). Immunohistologic study of bone marrow involvement in B-chronic lymphocytic leukemia. Blood.

[141] Granziero L., Ghia P., Circosta P., Gottardi D., Strola G., Geuna M., Montagna L., Piccoli P., Chilosi M., Caligaris-Cappio F. (2001). Survivin is expressed on CD40 stimulation and interfaces proliferation and apoptosis in B-cell chronic lymphocytic leukemia. Blood.

[142] Ghia P., Strola G., Granziero L., Geuna M., Guida G., Sallusto F., Ruffing N., Montagna L., Piccoli P., Chilosi M. (2002). Chronic lymphocytic leukemia B cells are endowed with the capacity to attract CD4+, CD40L+ T cells by producing CCL22. Eur J. Immunol.

[143] Patten P.E., Buggins A.G., Richards J., Wotherspoon A., Salisbury J., Mufti G.J., Hamblin T.J., Devereux S. (2008). CD38 expression in chronic lymphocytic leukemia is regulated by the tumor microenvironment. Blood.

[144] Hartmann E.M., Rudelius M., Burger J.A., Rosenwald A. (2016). CCL3 chemokine expression by chronic lymphocytic leukemia cells orchestrates the composition of the microenvironment in lymph node infiltrates. Leuk. Lymphoma.

[145] Zucchetto A., Tripodo C., Benedetti D., Deaglio S., Gaidano G., Del Poeta G., Gattei V. (2010). Monocytes/macrophages but not T lymphocytes are the major targets of the CCL3/CCL4 chemokines produced by CD38(+)CD49d(+) chronic lymphocytic leukaemia cells. Br. J. Haematol..

[146] Sivina M., Hartmann E., Kipps T.J., Rassenti L., Krupnik D., Lerner S., LaPushin R., Xiao L., Huang X., Werner L. (2011). CCL3 (MIP-1alpha) plasma levels and the risk for disease progression in chronic lymphocytic leukemia. Blood.

[147] Huergo-Zapico L., Acebes-Huerta A., Gonzalez-Rodriguez A.P., Contesti J., Gonzalez-Garcia E., Payer A.R., Villa-Alvarez M., Fernandez-Guizan A., Lopez-Soto A., Gonzalez S. (2014). Expansion of NK cells and reduction of NKG2D expression in chronic lymphocytic leukemia. Correlation with progressive disease. PLoS ONE.

[148] MacFarlane A.W.T., Jillab M., Smith M.R., Alpaugh R.K., Cole M.E., Litwin S., Millenson M.M., Al-Saleem T., Cohen A.D., Campbell K.S. (2017). NK cell dysfunction in chronic lymphocytic leukemia is associated with loss of the mature cells expressing inhibitory killer cell Ig-like receptors. Oncoimmunology.

[149] Maki G., Hayes G.M., Naji A., Tyler T., Carosella E.D., Rouas-Freiss N., Gregory S.A. (2008). NK resistance of tumor cells from multiple myeloma and chronic lymphocytic leukemia patients: Implication of HLA-G. Leukemia.

[150] Buechele C., Baessler T., Schmiedel B.J., Schumacher C.E., Grosse-Hovest L., Rittig K., Salih H.R. (2012). 4-1BB ligand modulates direct and Rituximab-induced NK-cell reactivity in chronic lymphocytic leukemia. Eur. J. Immunol..

[151] Xing D.X., Ramsay A.G., Robinson S., Bollard C.M., Shah N., Champlin R., Gribben J.G., Shpall E.J. (2011). Lenalidomide Treatment Enhances Immunological Synapse Formation of Cord Blood Natural Killer Cells with B Cells Derived From Chronic Lymphocytic Leukemia. Blood.

[152] Palmer S., Hanson C.A., Zent C.S., Porrata L.F., Laplant B., Geyer S.M., Markovic S.N., Call T.G., Bowen D.A., Jelinek D.F. (2008). Prognostic importance of T and NK-cells in a consecutive series of newly diagnosed patients with chronic lymphocytic leukaemia. Br. J. Haematol..

[153] Spaargaren M., Beuling E.A., Rurup M.L., Meijer H.P., Klok M.D., Middendorp S., Hendriks R.W., Pals S.T. (2003). The B cell antigen receptor controls integrin activity through Btk and PLCgamma2. J. Exp. Med..

[154] Ortolano S., Hwang I.Y., Han S.B., Kehrl J.H. (2006). Roles for phosphoinositide 3-kinases, Bruton’s tyrosine kinase, and Jun kinases in B lymphocyte chemotaxis and homing. Eur. J. Immunol..

[155] De Gorter D.J., Beuling E.A., Kersseboom R., Middendorp S., van Gils J.M., Hendriks R.W., Pals S.T., Spaargaren M. (2007). Bruton’s tyrosine kinase and phospholipase Cgamma2 mediate chemokine-controlled B cell migration and homing. Immunity.

[156] Chen S.S., Chang B.Y., Chang S., Tong T., Ham S., Sherry B., Burger J.A., Rai K.R., Chiorazzi N. (2016). BTK inhibition results in impaired CXCR4 chemokine receptor surface expression, signaling and function in chronic lymphocytic leukemia. Leukemia.

[157] Palma M., Krstic A., Pena Perez L., Berglof A., Meinke S., Wang Q., Blomberg K.E.M., Kamali-Moghaddam M., Shen Q., Jaremko G. (2018). Ibrutinib induces rapid down-regulation of inflammatory markers and altered transcription of chronic lymphocytic leukaemia-related genes in blood and lymph nodes. Br. J. Haematol.

[158] Maharaj K., Sahakian E., Pinilla-Ibarz J. (2017). Emerging role of BCR signaling inhibitors in immunomodulation of chronic lymphocytic leukemia. Blood Adv..

[159] Aguilar-Hernandez M.M., Blunt M.D., Dobson R., Yeomans A., Thirdborough S., Larrayoz M., Smith L.D., Linley A., Strefford J.C., Davies A. (2016). IL-4 enhances expression and function of surface IgM in CLL cells. Blood.

[160] Lundin J., Kimby E., Bergmann L., Karakas T., Mellstedt H., Osterborg A. (2001). Interleukin 4 therapy for patients with chronic lymphocytic leukaemia: A phase I/II study. Br. J. Haematol..

[161] Kuppers R. (2009). The biology of Hodgkin’s lymphoma. Nat. Rev. Cancer.

[162] Skinnider B.F., Mak T.W. (2002). The role of cytokines in classical Hodgkin lymphoma. Blood.

[163] Fischer M., Juremalm M., Olsson N., Backlin C., Sundstrom C., Nilsson K., Enblad G., Nilsson G. (2003). Expression of CCL5/RANTES by Hodgkin and Reed-Sternberg cells and its possible role in the recruitment of mast cells into lymphomatous tissue. Int. J. Cancer.

[164] Aldinucci D., Lorenzon D., Cattaruzza L., Pinto A., Gloghini A., Carbone A., Colombatti A. (2008). Expression of CCR5 receptors on Reed-Sternberg cells and Hodgkin lymphoma cell lines: Involvement of CCL5/Rantes in tumor cell growth and microenvironmental interactions. Int. J. Cancer.

[165] Marshall N.A., Christie L.E., Munro L.R., Culligan D.J., Johnston P.W., Barker R.N., Vickers M.A. (2004). Immunosuppressive regulatory T cells are abundant in the reactive lymphocytes of Hodgkin lymphoma. Blood.

[166] Ishida T., Ishii T., Inagaki A., Yano H., Komatsu H., Iida S., Inagaki H., Ueda R. (2006). Specific recruitment of CC chemokine receptor 4-positive regulatory T cells in Hodgkin lymphoma fosters immune privilege. Cancer Res..

[167] Gandhi M.K., Lambley E., Duraiswamy J., Dua U., Smith C., Elliott S., Gill D., Marlton P., Seymour J., Khanna R. (2006). Expression of LAG-3 by tumor-infiltrating lymphocytes is coincident with the suppression of latent membrane antigen-specific CD8+ T-cell function in Hodgkin lymphoma patients. Blood.

[168] Cader F.Z., Schackmann R.C.J., Hu X., Wienand K., Redd R., Chapuy B., Ouyang J., Paul N., Gjini E., Lipschitz M. (2018). Mass cytometry of Hodgkin lymphoma reveals a CD4(+) regulatory T-cell-rich and exhausted T-effector microenvironment. Blood.

[169] Aldinucci D., Olivo K., Lorenzon D., Poletto D., Gloghini A., Carbone A., Pinto A. (2005). The role of interleukin-3 in classical Hodgkin’s disease. Leuk. Lymphoma.

[170] Tanijiri T., Shimizu T., Uehira K., Yokoi T., Amuro H., Sugimoto H., Torii Y., Tajima K., Ito T., Amakawa R. (2007). Hodgkin’s reed-sternberg cell line (KM-H2) promotes a bidirectional differentiation of CD4+CD25+Foxp3+ T cells and CD4+ cytotoxic T lymphocytes from CD4+ naive T cells. J. Leukoc. Biol..

[171] Alvaro T., Lejeune M., Salvado M.T., Bosch R., Garcia J.F., Jaen J., Banham A.H., Roncador G., Montalban C., Piris M.A. (2005). Outcome in Hodgkin’s lymphoma can be predicted from the presence of accompanying cytotoxic and regulatory T cells. Clin. Cancer Res..

[172] Kelley T.W., Pohlman B., Elson P., Hsi E.D. (2007). The ratio of FOXP3+ regulatory T cells to granzyme B+ cytotoxic T/NK cells predicts prognosis in classical Hodgkin lymphoma and is independent of bcl-2 and MAL expression. Am. J. Clin. Pathol..

[173] Greaves P., Clear A., Owen A., Iqbal S., Lee A., Matthews J., Wilson A., Calaminici M., Gribben J.G. (2013). Defining characteristics of classical Hodgkin lymphoma microenvironment T-helper cells. Blood.

[174] Newcom S.R., Gu L. (1995). Transforming growth factor beta 1 messenger RNA in Reed-Sternberg cells in nodular sclerosing Hodgkin’s disease. J. Clin. Pathol..

[175] Chemnitz J.M., Driesen J., Classen S., Riley J.L., Debey S., Beyer M., Popov A., Zander T., Schultze J.L. (2006). Prostaglandin E2 impairs CD4+ T cell activation by inhibition of lck: Implications in Hodgkin’s lymphoma. Cancer Res..

[176] Yamamoto R., Nishikori M., Kitawaki T., Sakai T., Hishizawa M., Tashima M., Kondo T., Ohmori K., Kurata M., Hayashi T. (2008). PD-1-PD-1 ligand interaction contributes to immunosuppressive microenvironment of Hodgkin lymphoma. Blood.

[177] Roemer M.G., Advani R.H., Ligon A.H., Natkunam Y., Redd R.A., Homer H., Connelly C.F., Sun H.H., Daadi S.E., Freeman G.J. (2016). PD-L1 and PD-L2 Genetic Alterations Define Classical Hodgkin Lymphoma and Predict Outcome. J. Clin. Oncol..

[178] Reichel J., Chadburn A., Rubinstein P.G., Giulino-Roth L., Tam W., Liu Y., Gaiolla R., Eng K., Brody J., Inghirami G. (2015). Flow sorting and exome sequencing reveal the oncogenome of primary Hodgkin and Reed-Sternberg cells. Blood.

[179] Roemer M.G., Advani R.H., Redd R.A., Pinkus G.S., Natkunam Y., Ligon A.H., Connelly C.F., Pak C.J., Carey C.D., Daadi S.E. (2016). Classical Hodgkin Lymphoma with Reduced beta2M/MHC Class I Expression Is Associated with Inferior Outcome Independent of 9p24.1 Status. Cancer Immunol. Res..

[180] Roemer M.G.M., Redd R.A., Cader F.Z., Pak C.J., Abdelrahman S., Ouyang J., Sasse S., Younes A., Fanale M., Santoro A. (2018). Major Histocompatibility Complex Class II and Programmed Death Ligand 1 Expression Predict Outcome After Programmed Death 1 Blockade in Classic Hodgkin Lymphoma. J. Clin. Oncol..

[181] Koh Y.W., Jeon Y.K., Yoon D.H., Suh C., Huh J. (2016). Programmed death 1 expression in the peritumoral microenvironment is associated with a poorer prognosis in classical Hodgkin lymphoma. Tumour Biol..

[182] Hollander P., Kamper P., Smedby K.E., Enblad G., Ludvigsen M., Mortensen J., Amini R.M., Hamilton-Dutoit S., d’Amore F., Molin D. (2017). High proportions of PD-1(+) and PD-L1(+) leukocytes in classical Hodgkin lymphoma microenvironment are associated with inferior outcome. Blood Adv..

[183] Hollander P., Amini R.M., Ginman B., Molin D., Enblad G., Glimelius I. (2018). Expression of PD-1 and PD-L1 increase in consecutive biopsies in patients with classical Hodgkin lymphoma. PLoS ONE.

[184] Steidl C., Lee T., Shah S.P., Farinha P., Han G., Nayar T., Delaney A., Jones S.J., Iqbal J., Weisenburger D.D. (2010). Tumor-associated macrophages and survival in classic Hodgkin’s lymphoma. N. Engl. J. Med..

[185] Guo B., Cen H., Tan X., Ke Q. (2016). Meta-analysis of the prognostic and clinical value of tumor-associated macrophages in adult classical Hodgkin lymphoma. BMC Med..

[186] Carey C.D., Gusenleitner D., Lipschitz M., Roemer M.G.M., Stack E.C., Gjini E., Hu X., Redd R., Freeman G.J., Neuberg D. (2017). Topological analysis reveals a PD-L1-associated microenvironmental niche for Reed-Sternberg cells in Hodgkin lymphoma. Blood.

[187] Wotherspoon A.C., Ortiz-Hidalgo C., Falzon M.R., Isaacson P.G. (1991). Helicobacter pylori-associated gastritis and primary B-cell gastric lymphoma. Lancet.

[188] Wundisch T., Thiede C., Morgner A., Dempfle A., Gunther A., Liu H., Ye H., Du M.Q., Kim T.D., Bayerdorffer E. (2005). Long-term follow-up of gastric MALT lymphoma after Helicobacter pylori eradication. J. Clin. Oncol..

[189] Streubel B., Simonitsch-Klupp I., Mullauer L., Lamprecht A., Huber D., Siebert R., Stolte M., Trautinger F., Lukas J., Puspok A. (2004). Variable frequencies of MALT lymphoma-associated genetic aberrations in MALT lymphomas of different sites. Leukemia.

[190] Troppan K., Wenzl K., Neumeister P., Deutsch A. (2015). Molecular Pathogenesis of MALT Lymphoma. Gastroenterol. Res. Pract..

[191] Hussell T., Isaacson P.G., Crabtree J.E., Spencer J. (1993). The response of cells from low-grade B-cell gastric lymphomas of mucosa-associated lymphoid tissue to Helicobacter pylori. Lancet.

[192] Hussell T., Isaacson P.G., Crabtree J.E., Spencer J. (1996). Helicobacter pylori-specific tumour-infiltrating T cells provide contact dependent help for the growth of malignant B cells in low-grade gastric lymphoma of mucosa-associated lymphoid tissue. J. Pathol..

[193] Huynh M.Q., Wacker H.H., Wundisch T., Sohlbach K., Kim T.D., Krause M., Stabla K., Roth P., Fischbach W., Stolte M. (2008). Expression profiling reveals specific gene expression signatures in gastric MALT lymphomas. Leuk. Lymphoma.

[194] Knorr C., Amrehn C., Seeberger H., Rosenwald A., Stilgenbauer S., Ott G., Muller Hermelink H.K., Greiner A. (1999). Expression of costimulatory molecules in low-grade mucosa-associated lymphoid tissue-type lymphomas in vivo. Am. J. Pathol..

[195] Craig V.J., Cogliatti S.B., Arnold I., Gerke C., Balandat J.E., Wundisch T., Muller A. (2010). B-cell receptor signaling and CD40 ligand-independent T cell help cooperate in Helicobacter-induced MALT lymphomagenesis. Leukemia.

[196] Munari F., Lonardi S., Cassatella M.A., Doglioni C., Cangi M.G., Amedei A., Facchetti F., Eishi Y., Rugge M., Fassan M. (2011). Tumor-associated macrophages as major source of APRIL in gastric MALT lymphoma. Blood.

[197] Vose J.M. (2017). Mantle cell lymphoma: 2017 update on diagnosis, risk-stratification, and clinical management. Am. J. Hematol..

[198] Wang M., Sun L., Qian J., Han X., Zhang L., Lin P., Cai Z., Yi Q. (2009). Cyclin D1 as a universally expressed mantle cell lymphoma-associated tumor antigen for immunotherapy. Leukemia.

[199] Bea S., Valdes-Mas R., Navarro A., Salaverria I., Martin-Garcia D., Jares P., Gine E., Pinyol M., Royo C., Nadeu F. (2013). Landscape of somatic mutations and clonal evolution in mantle cell lymphoma. Proc. Natl. Acad. Sci. USA.

[200] Medina D.J., Goodell L., Glod J., Gelinas C., Rabson A.B., Strair R.K. (2012). Mesenchymal stromal cells protect mantle cell lymphoma cells from spontaneous and drug-induced apoptosis through secretion of B-cell activating factor and activation of the canonical and non-canonical nuclear factor kappaB pathways. Haematologica.

[201] Ek S., Bjorck E., Hogerkorp C.M., Nordenskjold M., Porwit-MacDonald A., Borrebaeck C.A. (2006). Mantle cell lymphomas acquire increased expression of CCL4, CCL5 and 4-1BB-L implicated in cell survival. Int. J. Cancer.

[202] Nygren L., Wasik A.M., Baumgartner-Wennerholm S., Jeppsson-Ahlberg A., Klimkowska M., Andersson P., Buhrkuhl D., Christensson B., Kimby E., Wahlin B.E. (2014). T-cell levels are prognostic in mantle cell lymphoma. Clin. Cancer Res..

[203] Zhang X.Y., Xu J., Zhu H.Y., Wang Y., Wang L., Fan L., Wu Y.J., Li J.Y., Xu W. (2016). Negative prognostic impact of low absolute CD4(+) T cell counts in peripheral blood in mantle cell lymphoma. Cancer Sci..

[204] Muenst S., Hoeller S., Willi N., Dirnhofera S., Tzankov A. (2010). Diagnostic and prognostic utility of PD-1 in B cell lymphomas. Dis. Markers.

[205] Wang L., Qian J., Lu Y., Li H., Bao H., He D., Liu Z., Zheng Y., He J., Li Y. (2013). Immune evasion of mantle cell lymphoma: Expression of B7-H1 leads to inhibited T-cell response to and killing of tumor cells. Haematologica.

[206] Harrington B.K., Wheeler E., Hornbuckle K., Y Shana’ah A., Youssef Y., Smith L., Hassan Ii Q., Klamer B., Zhang X., Long M. (2019). Modulation of immune checkpoint molecule expression in mantle cell lymphoma. Leuk. Lymphoma.

[207] Song K., Herzog B.H., Sheng M., Fu J., McDaniel J.M., Chen H., Ruan J., Xia L. (2013). Lenalidomide inhibits lymphangiogenesis in preclinical models of mantle cell lymphoma. Cancer Res..

[208] Pham L.V., Vang M.T., Tamayo A.T., Lu G., Challagundla P., Jorgensen J.L., Rollo A.A., Ou Z., Zhang L., Wang M. (2015). Involvement of tumor-associated macrophage activation in vitro during development of a novel mantle cell lymphoma cell line, PF-1, derived from a typical patient with relapsed disease. Leuk. Lymphoma.

[209] Koh Y.W., Shin S.J., Park C., Yoon D.H., Suh C., Huh J. (2014). Absolute monocyte count predicts overall survival in mantle cell lymphomas: Correlation with tumour-associated macrophages. Hematol. Oncol..

[210] Papin A., Tessoulin B., Bellanger C., Moreau A., Le Bris Y., Maisonneuve H., Moreau P., Touzeau C., Amiot M., Pellat-Deceunynck C. (2019). CSF1R and BTK inhibitions as novel strategies to disrupt the dialog between mantle cell lymphoma and macrophages. Leukemia.

[211] Sagiv-Barfi I., Kohrt H.E., Czerwinski D.K., Ng P.P., Chang B.Y., Levy R. (2015). Therapeutic antitumor immunity by checkpoint blockade is enhanced by ibrutinib, an inhibitor of both BTK and ITK. Proc. Natl. Acad. Sci. USA.

[212] Ansell S.M., Hurvitz S.A., Koenig P.A., LaPlant B.R., Kabat B.F., Fernando D., Habermann T.M., Inwards D.J., Verma M., Yamada R. (2009). Phase I study of ipilimumab, an anti-CTLA-4 monoclonal antibody, in patients with relapsed and refractory B-cell non-Hodgkin lymphoma. Clin. Cancer Res..

[213] Lesokhin A.M., Ansell S.M., Armand P., Scott E.C., Halwani A., Gutierrez M., Millenson M.M., Cohen A.D., Schuster S.J., Lebovic D. (2016). Nivolumab in Patients With Relapsed or Refractory Hematologic Malignancy: Preliminary Results of a Phase Ib Study. J. Clin. Oncol..

[214] Ansell S.M., Minnema M.C., Johnson P., Timmerman J.M., Armand P., Shipp M.A., Rodig S.J., Ligon A.H., Roemer M.G.M., Reddy N. (2019). Nivolumab for Relapsed/Refractory Diffuse Large B-Cell Lymphoma in Patients Ineligible for or Having Failed Autologous Transplantation: A Single-Arm, Phase II Study. J. Clin. Oncol..

[215] Younes A., Brody J., Carpio C., Lopez-Guillermo A., Ben-Yehuda D., Ferhanoglu B., Nagler A., Ozcan M., Avivi I., Bosch F. (2019). Safety and activity of ibrutinib in combination with nivolumab in patients with relapsed non-Hodgkin lymphoma or chronic lymphocytic leukaemia: A phase 1/2a study. Lancet Haematol..

[216] Zinzani P.L., Ribrag V., Moskowitz C.H., Michot J.M., Kuruvilla J., Balakumaran A., Zhang Y., Chlosta S., Shipp M.A., Armand P. (2017). Safety and tolerability of pembrolizumab in patients with relapsed/refractory primary mediastinal large B-cell lymphoma. Blood.

[217] Chan T.S., Khong P.L., Kwong Y.L. (2016). Pembrolizumab and lenalidomide induced remission in refractory double-hit lymphoma. Ann. Hematol..

[218] Armand P., Nagler A., Weller E.A., Devine S.M., Avigan D.E., Chen Y.B., Kaminski M.S., Holland H.K., Winter J.N., Mason J.R. (2013). Disabling immune tolerance by programmed death-1 blockade with pidilizumab after autologous hematopoietic stem-cell transplantation for diffuse large B-cell lymphoma: Results of an international phase II trial. J. Clin. Oncol..

[219] Advani R., Flinn I., Popplewell L., Forero A., Bartlett N.L., Ghosh N., Kline J., Roschewski M., LaCasce A., Collins G.P. (2018). CD47 Blockade by Hu5F9-G4 and Rituximab in Non-Hodgkin’s Lymphoma. N. Engl. J. Med..

[220] O’Mahony D., Morris J.C., Quinn C., Gao W., Wilson W.H., Gause B., Pittaluga S., Neelapu S., Brown M., Fleisher T.A. (2007). A pilot study of CTLA-4 blockade after cancer vaccine failure in patients with advanced malignancy. Clin. Cancer Res..

[221] Nastoupil L., Westin J.R., Fowler N.H., Fanale M.A., Samaniego F., Oki Y., Dsouza L., Obi C., Cao J.J., Cheng X.Y. (2017). High Complete Response Rates with Pembrolizumab in Combination with Rituximab in Patients with Relapsed Follicular Lymphoma: Results of an Open-Label, Phase II Study. Blood.

[222] Ding W., LaPlant B.R., Call T.G., Parikh S.A., Leis J.F., He R., Shanafelt T.D., Sinha S., Le-Rademacher J., Feldman A.L. (2017). Pembrolizumab in patients with CLL and Richter transformation or with relapsed CLL. Blood.

[223] Ansell S.M., Lesokhin A.M., Borrello I., Halwani A., Scott E.C., Gutierrez M., Schuster S.J., Millenson M.M., Cattry D., Freeman G.J. (2015). PD-1 blockade with nivolumab in relapsed or refractory Hodgkin’s lymphoma. N. Engl. J. Med..

[224] Armand P., Engert A., Younes A., Fanale M., Santoro A., Zinzani P.L., Timmerman J.M., Collins G.P., Ramchandren R., Cohen J.B. (2018). Nivolumab for Relapsed/Refractory Classic Hodgkin Lymphoma After Failure of Autologous Hematopoietic Cell Transplantation: Extended Follow-Up of the Multicohort Single-Arm Phase II CheckMate 205 Trial. J. Clin. Oncol..

[225] Cheson B.D., Ansell S., Schwartz L., Gordon L.I., Advani R., Jacene H.A., Hoos A., Barrington S.F., Armand P. (2016). Refinement of the Lugano Classification lymphoma response criteria in the era of immunomodulatory therapy. Blood.

[226] Merryman R.W., Kim H.T., Zinzani P.L., Carlo-Stella C., Ansell S.M., Perales M.A., Avigdor A., Halwani A.S., Houot R., Marchand T. (2017). Safety and efficacy of allogeneic hematopoietic stem cell transplant after PD-1 blockade in relapsed/refractory lymphoma. Blood.

[227] Herrera A.F., Moskowitz A.J., Bartlett N.L., Vose J.M., Ramchandren R., Feldman T.A., LaCasce A.S., Ansell S.M., Moskowitz C.H., Fenton K. (2018). Interim results of brentuximab vedotin in combination with nivolumab in patients with relapsed or refractory Hodgkin lymphoma. Blood.

[228] Advani R.H., Moskowitz A.J., Bartlett N.L., Vose J.M., Ramchandren R., Feldman T.A., LaCasce A.S., Christian B.A., Ansell S.M., Moskowitz C.H. (2018). Phase 1/2 Study of Brentuximab Vedotin in Combination with Nivolumab in Patients with Relapsed or Refractory Classic Hodgkin Lymphoma: Part 3 (Concurrent Dosing) Results and Updated Progression-Free Survival Results from Parts 1 and 2 (Staggered Dosing). Blood.

[229] Armand P., Shipp M.A., Ribrag V., Michot J.M., Zinzani P.L., Kuruvilla J., Snyder E.S., Ricart A.D., Balakumaran A., Rose S. (2016). Programmed Death-1 Blockade with Pembrolizumab in Patients with Classical Hodgkin Lymphoma After Brentuximab Vedotin Failure. J. Clin. Oncol..

[230] Chen R., Zinzani P.L., Fanale M.A., Armand P., Johnson N.A., Brice P., Radford J., Ribrag V., Molin D., Vassilakopoulos T.P. (2017). Phase II Study of the Efficacy and Safety of Pembrolizumab for Relapsed/Refractory Classic Hodgkin Lymphoma. J. Clin. Oncol..

[231] Armand P., Chen Y.B., Redd R.A., Joyce R.M., Bsat J., Jeter E., Merryman R.W., Coleman K.C., Dahi P.B., Nieto Y. (2019). PD-1 Blockade with Pembrolizumab for Classical Hodgkin Lymphoma after Autologous Stem Cell Transplantation. Blood.

[232] Bashey A., Medina B., Corringham S., Pasek M., Carrier E., Vrooman L., Lowy I., Solomon S.R., Morris L.E., Holland H.K. (2009). CTLA4 blockade with ipilimumab to treat relapse of malignancy after allogeneic hematopoietic cell transplantation. Blood.

[233] Pianko M.J., Liu Y., Bagchi S., Lesokhin A.M. (2017). Immune checkpoint blockade for hematologic malignancies: A review. Stem Cell Investig..

